# Responses of Plant Reproductive Phenology to Winter-Biased Warming in an Alpine Meadow

**DOI:** 10.3389/fpls.2020.534703

**Published:** 2020-09-04

**Authors:** Xiaoli Hu, Wenlong Zhou, Shucun Sun

**Affiliations:** Department of Biology, School of Life Sciences, Nanjing University, Nanjing, China

**Keywords:** experimental warming, flowering phenology, fruiting phenology, plant trait, root to shoot ratio, seed size, plant height, alpine meadow

## Abstract

Climate warming is often seasonally asymmetric with a higher temperature increase toward winters than summers. However, the effect of winter-biased warming on plant reproductive phenology has been seldom investigated under natural field conditions. The goal of this study was to determine the effects of winter-biased warming on plant reproductive phenologies. In an alpine meadow of Tibetan Plateau, we deployed six large (15 m × 15 m × 2.5 m height) open top chambers (three warmed chambers and three non-warmed chambers) to achieve winter-biased warming (i.e., a small increase in annual mean temperature with a greater increase towards winter than summer). We investigated three phenophases (onset and offset times and duration) for both the flowering and fruiting phenologies of 11 common species in 2017 and 8 species in 2018. According to the vernalization theory, we hypothesized that mild winter-biased warming would delay flowering and fruiting phenologies. The data indicated that the phenological responses to warming were species-specific (including positive, neutral, and negative responses), and the number of plant species advancing flowering (by averagely 4.5 days) and fruiting onset times (by averagely 3.6 days) was higher than those delaying the times. These changes were inconsistent with the vernalization hypothesis (i.e. plants need to achieve a threshold of chilling before flowering) alone, but can be partly explained by the accumulated temperature hypothesis (i.e. plants need to achieve a threshold of accumulative temperature before flowering) and/or the overtopping hypothesis (i.e. plants need to reach community canopy layer before flowering). The interspecific difference in the response of reproductive phenology could be attributed to the variation in plant traits including plant height growth, the biomass ratio of root to shoot, and seed mass. These results indicate that a mild winter-biased warming may trigger significant change in plant reproductive phenology in an alpine meadow.

## Introduction

Global mean surface temperature has increased by 0.8–1.2°C from 1950 to 2017 with a rate of 0.1–0.3°C per decade (IPCC, 2018), and it is predicted to increase (by either 0.3–1.7°C according to RCP 2.6 (Representative Concentration Pathway), and 2.6–4.8°C according to RCP 8.5) by the end of the 21^st^ century (IPCC, 2014). Moreover, the magnitude of climate warming is heterogeneous both spatially and temporally, and is generally greater at higher latitudes (e.g. the arctic regions; Meredith et al., 2019) and higher altitudes (Hock et al., 2019). The magnitude of climate warming is also greater in winter than summer and at night than during the day (IPCC, 2007; IPCC, 2014). Typically, the Tibetan Plateau has experienced a greater than global average increase (0.16°C per decade; IPCC, 2018) in mean annual temperature (0.25°C per decade), with 0.30°C per decade in winter, greater than that of summer (0.20°C per decade) since the 1960s (Liu and Chen, 2000).

Numerous studies have shown that climate warming affects almost all aspects of biological systems. One of the most extensive studied aspect is plant phenology (Arft et al., 1999; Dunne et al., 2003; Sherry et al., 2007; Post et al., 2008; Dorji et al., 2013; Meng et al., 2019; Jabis et al., 2020), since warming-induced changes in plant phenology may affect species interactions, ecosystem nutrient cycling, and energy flow (CaraDonna et al., 2014). Climate warming is often simulated by manipulative experiments using open top chambers or infrared heaters in different terrestrial biomes (Marion et al., 1997; Arft et al., 1999; Sherry et al., 2007; Post et al., 2008; Dorji et al., 2013). These warming experiments usually elevate annual mean temperature by 1.2–5°C, which is generally greater than the predicted temperature changes at a century scale (e.g., Marion et al., 1997; Post et al., 2008; Dorji et al., 2013). However, it is often suggested that long-term and mild warming experiments are more likely to provide data reflective of more realistic conditions and results. Importantly, artificial warming often archives a higher temperature increase in summers than in winters because infrared heaters are usually turned off or less effective in winters (Kimball, 2005; Zhou et al., 2019), and because open top chambers often result in higher temperature increases in summers than winters (Marion et al., 1997; Post et al., 2008; Dorji et al., 2013).

Although studies have substantially addressed plant responses to climate warming in the growing (summer) season, winter warming is a key driver of plant performance in terrestrial ecosystems, especially in cold regions (Williams et al., 2015; Shen et al., 2016). For example, long-term remote sensing data from Tibetan Plateau show that an increase in winter temperatures would substantially advance the start date of vegetation greening (Zhang et al., 2013; Shen et al., 2016). It is surprising, therefore, that a mild winter-biased warming has been scarcely employed to examine phenological responses, especially because it is predicted to be a realistic future condition.

Winter-biased warming may have different effects on plant phenology as suggested by three different hypotheses. For example, winter warming is predicted to decrease the strength of chilling to delay the timing of leafing, flowering, and fruiting in many species (i.e. the vernalization hypothesis; Körner and Basler, 2010). The vernalization hypothesis is supported by the delay of the spring greening of vegetation in Tibetan grasslands, as recorded by remote sensing images (Yu et al., 2010). Winter warming is also predicted to enhance accumulative temperature to reach an earlier threshold for flowering, and therefore it may advance plant reproductive phenology (i.e. the accumulated temperature hypothesis; Harrington and Gould, 2015). Winter warming may also facilitate plant growth by accelerating litter decomposition (Bernier et al., 1981) and hence advance flowering phenology by allowing plants to achieve an earlier canopy layer, as suggested by the overtopping hypothesis positing that plants tend to flower when they reach their canopy layer (Wesselingh et al., 1997; Jacquemyn et al., 2010). The overtopping hypothesis has been supported by many studies (Pfeifer et al., 2006; Zhou et al., 2019). Thus, predictions based on different mechanisms are not consistent about whether winter warming will advance or delay plant reproductive phenology.

Moreover, it is not clear whether all co-occurring species will show the same response to warming (Sherry et al., 2007; Dorji et al., 2013). This is often explained by species-specific functional traits (Dorji et al., 2013; Zhou et al., 2019). For example, warming may affect growth in plant height differentially (Baruah et al., 2017), such that the time at which a plant reaches canopy spread and flowering or fruiting differs among species according to the overtopping hypothesis. Moreover, according to the seed size-time hypothesis, which proposes that perennial species with large seeds require a longer time to develop mature fruits, large seeded-species will tend to flower and fruit earlier and hence have a longer development time (Bolmgren and Cowan, 2008; Du and Qi, 2010). Consequently, if warming induced an advance of flowering or fruiting onset time in one species, it would advance the species’ flowering or fruiting offset time (if seed size is unchanged by warming). In addition, plants often differ in root depth and root/shoot mass ratio (R/S), which may mediate the response of plant phenology to warming. The plants with shallow roots or lower R/S may be more sensitive (relative to the species with deeper roots or higher R/S) to warming as a consequence of soil moisture deficits (Passioura, 1983; Dorji et al., 2013), and hence the species with contrasting root depths and R/S may differ in their phenological responses to warming.

To fully understand the effect of winter-biased mild warming on plant phenology, we investigated the onset and offset times of reproductive phenology (flowering and fruiting) for 11 herbaceous species growing in both (artificially) warmed and non-warmed open top chambers for two consecutive years in a Tibetan meadow. The objectives of this study were 1) to determine whether the phenological response to artificial warming is consistent with the vernalization hypothesis, the accumulated temperature hypothesis, or the overtopping hypothesis, and 2) to test whether interspecific differences in the growth rates of plant height, root/shoot mass ratio, and seed size accounted for any of the variation in the phenological responses among the study species.

## Materials and Methods

### Study Site

This research was conducted in Hongyuan County, Sichuan province, China (32°48’N, 102°33’E), which is in the eastern Tibetan Plateau with an altitude of 3,500 m a.s.l. (**Figure S1A**). The climate is characterized by long, cold winter, short spring and autumn, and a cool mild summer. According to data collected at Hongyuan County Climate Station (5 km away from the study site) from 1970 to 2016, annual mean temperature is 1.7°C, with maximum and minimum monthly means of 11.1°C and −9.3°C observed in July and January. Mean annual precipitation is 756 mm (including 73 mm snow precipitation), over 80% of which falls during the growing season from May to September (Cao et al., 2018). Relative to 1970–2000, mean annual temperature has increased by 0.97°C during 2001–2016, with a higher increase in non-growing seasons (October to April, 1.04°C) than growing seasons (May to September, 0.88°C).

The pasture has been intensively grazed by livestock (e.g. yak *Bos grunniens*) for decades. The studied meadow is dominated by an assemblage of forbs (*Saussurea nigrescens*, *Polygonum viviparum*, *Potentilla anserine*, *Trollius farreri*, *Thalictrum alpinum*, and *Anemone trullifolia* var. *linearis*), sedges (*Kobresia setchwanensis* and *Carex* spp.), and grasses (*Deschampsia caespitosa*, *Festuca ovina* and *Elymus nutans*). Vegetation coverage of the meadow is more than 90%, and average plant height is ~30 cm (Wu et al., 2011). Owing to the diverse plant species (Xiang et al., 2009), the arthropod species, like pollinators (Hu et al., 2019), herbivores (Xi et al., 2013), as well as dung decomposers (Wu et al., 2011) are diverse in the meadow.

### Experimental Design

In October of 2014, six 15 × 15 × 2.5 m (height) open top chambers (OTCs) were randomly deployed in a fenced (non-grazed area) flat area of about 1.0 ha. The sides of all OTCs were covered with thin (less than 0.1 mm) steel screen with a mesh size of 0.2 × 0.2 mm. Three of the OTCs were additionally covered with transparent tempered glass (δ8). The roof of these three OTCs was discontinuously covered by 0.15 × 0.3 m (width) transparent glass strips, with a 0.6 m space between neighboring strips (**Figures S1B, C**). Each OTC was sunk 1 m into the soil, and along the OTC sides, steel screen (with a mesh size of 0.6 × 0.6 mm) was also sunk 1 m into the soil to prevent rodents from entering (**Figure S1B**). In mid-July of 2018, the transparency of the transparent tempered glass was on average (94.4%, N = 45) under full light conditions, slightly lower than that of the steel screen (97.9%, N = 45). We refer to the three OTCs with transparent tempered glass as warmed chambers and the other three as ambient, control, non-warmed chambers (**Figure S1D**).

### Microclimate Measurements

In each chamber, HOBO temperature sensors (HOBO PRO, Onset Computer Corporation, USA) were used to monitor air temperature (*T_i_*) at 30 cm above ground level. The HOBO MX2301A sensors (Onset Computer Corporation, USA) were deployed in a pair of chambers (one for warmed and the other for non-warmed) to monitor air relative humidity (*M_i_*) at 30 cm above ground level. The air vapor pressure deficit (*VPD*) was calculated using the following equation (Richard et al., 1998).

$$VPD=0.611\;exp\left(\frac{17.27\times Ti}{Ti+237.3}\right)\times (1-\frac{Mi}{100})$$

Any abnormal microclimate values due to sensor malfunctions were removed from the data set. Soil temperature and moisture (at 5 cm) were monitored (using Watchdog2000, Spectrum Technologies, Inc., USA) for a pair of chambers (one for warmed and the other for non-warmed) since 2015. Data were sampled at 1-hour intervals.

### Phenological Measurements

During the growing season of 2017 and 2018, eleven common species were chosen for phenological monitoring. They consisted of eleven species (*A. trullifolia* var. *linearis*, *T. alpinum*, *T. farreri*, *Anemone rivularis*, *Delphinium caeruleum*, *Anaphalis flavescens*, *S. nigrescens*, *P. viviparum*, *Potentilla discolor*, *Halenia elliptica*, and *Gentianopsis paludosa*). In each study year, we randomly selected and tagged 10–30 individuals for each species in each chamber (if available) before the occurrence of flower buds. For each tagged individual, the flowers or capitula were weekly counted and each of them was given a phenological score following Price and Waser (1998). Six phenological stages were recorded, including unopened buds (stage 1), open flowers (stage 2, stamens are visible), old flowers (stage 3, petals or stamens are withering), initialed fruit (stage 4, petals abscised but ovaries unexpanded), expanding fruit (stage 5, enlarged fruit), and dehisced fruit (stage 6). “Stage 6” was recorded when fruits dehisced (*T. farreri*, *D. caeruleum*, *P. discolor*, *H. elliptica*, and *G. paludosa*), and fallen (*A. trullifolia* var. *linearis*, *T. alpinum*, *A. rivularis*, and *P. viviparum*), or pappuses became fluffy (*A. flavescens* and *S. nigrescens*). For each census, an unweighted phenological score was calculated by averaging the stages present on each individual (Price and Waser, 1998; Dunne et al., 2003; Sherry et al., 2007). To reduce the variability among individual observations of phenological stages, we derived phenological variables by fitting linear regression to the sequence of phenological scores for each observed individual as a function of the day of the year for each species and for each year. Regressions were performed only with individuals for which at least four phenological scores were observed throughout the reproductive period. The 1430 individual regressions showed statistically significant fits (average *r^2^* = 0.93 ± 0.002 [1SE]; maximum *r^2^* = 1, minimum *r^2^* = 0.54; *P* < 0.05).

Using the regression equations, we calculated the following phenological variables for each plant: flowering onset time (stage 2), flowering offset time (stage 3.5), fruiting onset time (stage 4), and fruiting offset time (stage 6). “Duration of flowering” refers to the estimated time to progress from stage 2 to stage 3.5, and “duration of fruiting” refers to the number of days for an individual to progress from stage 4 to stage 6. Because plant abundance varied among years and chambers, only 8 species were available for analysis in 2018.

### Plant Traits

Typical plant traits assumed to be relevant to plant phenology were measured for this study, including plant height, root/shoot ratio, and (individual) seed mass. Plant height at the onset time of flowering (Hf), the distance from ground-surface to stem tips, were recorded when the first open flower was observed for each tagged individual (except for *A. trullifolia* var. *linearis*, whose Hf was missed in 2018). The other traits including R/S and seed mass were measured outside chambers. More than 20 fruiting plants were randomly selected in the field and then soaked in water to remove the residual soil. Each plant was dissected into belowground parts (roots) and aboveground parts (shoots). Both roots and shoots were weighted after drying for 72 h at 75°C. In addition, one mature fruit was sampled from each plant to cunt and weigh viable seeds. Seed mass was calculated as the total seed mass divided by sound seed number. Finally, root/shoot mass ratios (R/S) and seed mass were averaged for each species (except for *T. alpinum*, whose seed mass was not measured).

### Statistical Analysis

A series of generalized linear mixed models (GLMMs) was used to test the effects of warming (non-warmed vs. warmed) on reproductive phenologies and plant height for each species. In each model, warming (eight species were investigated in both study years), year, and species were set as fixed factors, and “OTC” was set as a random factor, with “individual” nested within “OTC”. Moreover, GLMMs were used to test the effects of warming on the six reproductive phenologies for each species, where warming and year were set as fixed factors, and “OTC” was set as a random factor, with “individual” nested within “OTC”. GLMMs were performed using the package “lme4” (Bates et al., 2015) and “lmerTest” (Kuznetsova et al., 2017), respectively.

We also determined whether a cross-species relationship between phenological changes and species traits (including Hf, R/S and seed size) existed using linear regressions (by “*lm*”). The phenological changes, as well as changes in Hf were quantified as the relative change intensity (Ri) for each species following the protocols of Armas et al. (2004), i.e., calculated as (P_w_ − P_n_)/(P_w_ + P_n_), where P_w_ and P_n_ were the observed phenologies/Hf in the warmed and non-warmed treatments, respectively. The index Ri had defined limits [−1,1]. Negative values indicated an advance in the phenological events or lower height. All observed dates were converted to Julian dates (days since Jan. 1st). The relationship between phenological changes and plant traits in 2017 were analyzed because not all species were observed in 2018. All analyses were conducted using R 3.5.3 (R Core Team, 2019).

## Results

### Microclimate Conditions

Measurements over a span of four years showed that the mean annual temperature was 0.3–0.5°C higher at 30 cm aboveground, 0.2–0.5°C at the 5 cm soil depth in the warmed OTCs than in the non-warmed OTCs (**Table S1**, **Figures S2 and S3**). The mean temperature was 0.4–0.6°C higher at 30 cm above the soil surface, and 0.8–1.1°C higher at the 5 cm soil depth in warmed chambers than non-warmed chambers in the non-growing season. During the growing season, the increased temperature was 0.03–0.47°C and −0.2–0.8°C higher at the soil surface and at the 5 cm soil depth in the warmed chambers, respectively (**Table S1**). Temperature increase was more pronounced in winter than in summer, and it was statistically significant at night (18:00–8:00) but not during the daytime (**Figures S4 and S5**). The vapor pressure deficit was 2.6–3.7% higher in the warmed than the non-warmed OTCs in 2018 (**Figure S6**). In addition, the soil moisture was 2–3% VWC (percent in volume water content) higher at the 5 cm soil depth in the non-warmed than in the warmed OTCs (**Figure S7**).

### Phenological Response to the Warming

**Figure 1:**
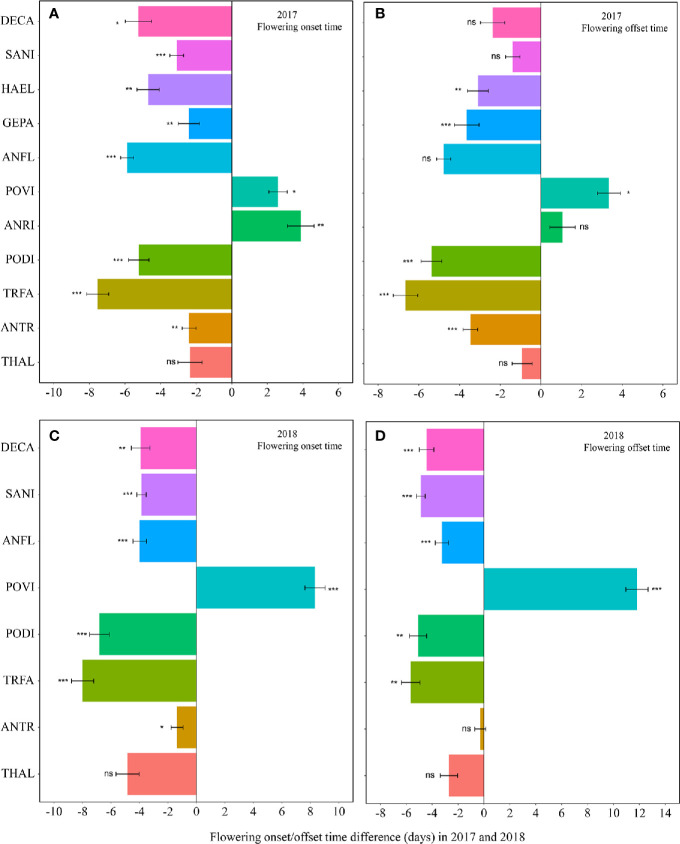
Changes in the flowering onset time (**A**, 2017; **C**, 2018), and flowering offset time (**B**, 2017; **D**, 2018) (in days) between the non-warmed and warmed chambers from 2017 to 2018. Species are listed in the order from the earliest flowering species *Thalictrum alpinum* to the latest flowering species *Delphinium caeruleum*. A negative value indicates the warming-induced advance in flowering onset time and offset time, while a positive value indicates the warming-induced delay in the phenologies. Bars indicate the mean ± SE for each bar. The difference in each phenology is determined by generalized linear mixed models (GLMMs). *P < 0.05; **P < 0.01; ***P < 0.001; ns, non-significant. THAL, *Thalictrum alpinum*; ANTR, *Anemone trullifolia* var. *linearis*; TRFA, *Trollius farreri*; PODI, *Potentilla discolor*; ANRI, *Anemone rivularis*; POVI, *Polygonum viviparum*; ANFL, *Anaphalis flavescens*; GEPA, *Gentianopsis paludosa*; HAEL, *Halenia elliptica*; SANI, *Saussurea nigrescens* and DECA, *Delphinium caeruleum*.

**Figure 2:**
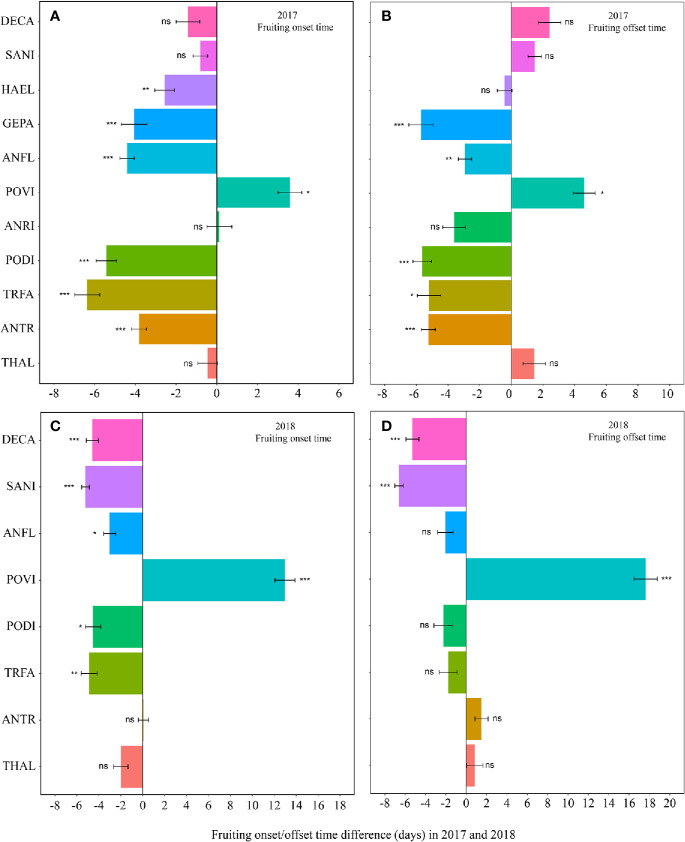
Changes in the fruiting onset time (**A**, 2017; **C**, 2018), and fruiting offset time (**B**, 2017; **D**, 2018) (in days) between the non-warmed and warmed chambers from 2017 to 2018. Species are listed in the order from the earliest flowering species *Thalictrum alpinum* to the latest flowering species *Delphinium caeruleum*. A negative value indicates the warming-induced advance in fruiting onset time and offset time, while a positive value indicates the warming-induced delay in the phenologies. Bars indicate the mean ± SE for each bar. The difference in each phenology is determined by generalized linear mixed models (GLMMs). *P < 0.05; **P < 0.01; ***P < 0.001; ns, non-significant. The abbreviation of species names is same as **Figure 1**.

Warming had a significant effect on the onset time of flowering and fruiting (**Table 1**, **Table S3**). Warming advanced flowering onset time for most species in both study years (significantly in 8 out of 11 species; with two exceptions in 2017 and one in 2018), with an average of 4.5 days (**Figures 1A, C**, **Tables S2 and S3**), but warming delayed the onset time of flowering and fruiting for *A. rivularis*, and *P. viviparum* in both 2017 and 2018 (**Figures 1A, C**, **Tables S2 and S3**). In contrast, experimental warming advanced fruiting onset time in 9 out of 11 species (significantly in 6 species) in 2017 and 5 out of 8 species in 2018, with an average of 3.6 days (**Figures 2A, C**, **Tables S2 and S3**), but delayed fruiting for *P. viviparum* in both 2017 and 2018.

**Table 1:** Summary of the GLMMs analysis of variance of the six phenological events (onset, offset, duration of flowering and fruiting) for two years.

	Source	Numerator DF	Denominator DF	*F*	*P*
**Flowering onset time**	Warming (W)	1	1256	91.0448	<0.0001***
	Year (Y)	1	1259.2	127.1054	<0.0001***
	Species (S)	7	1257.2	3298.0034	<0.0001***
	W: Y	1	1252.6	0.7007	0.402
	W: S	7	1255	13.6422	<0.0001***
	Y: S	7	1254.1	27.7809	<0.0001***
	W: Y: S	7	1254	1.5603	0.143
**Flowering offset time**	Warming (W)	1	1251.7	51.5803	<0.0001***
	Year (Y)	1	1252.2	82.2772	<0.0001***
	Species (S)	7	1252.1	3553.4587	<0.0001***
	W: Y	1	1248.1	1.9975	0.158
	W: S	7	1250.5	19.4363	<0.0001***
	Y: S	7	1248	13.6761	<0.0001***
	W: Y: S	7	1249.4	4.8524	<0.0001***
**Fruiting onset time**	Warming (W)	1	1251.2	31.7359	<0.0001***
	Year (Y)	1	1249	54.551	<0.0001***
	Species (S)	7	1251.1	2980.1729	<0.0001***
	W: Y	1	1248.1	2.1629	0.142
	W: S	7	1250	17.9819	<0.0001***
	Y: S	7	1247.5	18.0017	<0.0001***
	W: Y: S	7	1249	5.8225	<0.0001***
**Fruiting offset time**	Warming (W)	1	1253.6	0.6824	0.409
	Year (Y)	1	1252.6	3.6645	0.056.
	Species (S)	7	1253.1	1065.1179	<0.0001***
	W: Y	1	1252.7	1.7263	0.189
	W: S	7	1253.2	9.3641	<0.0001***
	Y: S	7	1254	41.4065	<0.0001***
	W: Y: S	7	1252.6	6.3516	<0.0001***
**Flowering duration**	Warming (W)	1	1253	23.8427	<0.0001***
	Year (Y)	1	1235.4	23.7434	<0.0001***
	Species (S)	7	1227.7	182.9706	<0.0001***
	W: Y	1	1253	0.4602	0.498
	W: S	7	1253.2	1.4248	0.191
	Y: S	7	1259	61.7383	<0.0001***
	W: Y: S	7	1252.6	3.7029	0.0006***
**Fruiting duration**	Warming (W)	1	1253	23.8427	<0.0001***
	Year (Y)	1	1235.4	23.7434	<0.0001***
	Species (S)	7	1227.7	182.9706	<0.0001***
	W: Y	1	1253	0.4602	0.498
	W: S	7	1253.2	1.4248	0.191
	Y: S	7	1259	61.7383	<0.0001***
	W: Y: S	7	1252.6	3.7029	0.0006***

The eight species (Thalictrum alpinum; ANTR, Anemone trullifolia var. linearis; TRFA, Trollius farreri; PODI, Potentilla discolor; POVI, Polygonum viviparum; ANFL, Anaphalis flavescens; SANI, Saussurea nigrescens and DECA, Delphinium caeruleum) observed in consecutive years were included.***P < 0.001.

The effect of warming was also significant on the offset time of flowering, but was not significant on fruiting offset time (**Table 1**). Warming advanced flowering offset time by averagely 3.6 days for all the species, with the exceptions of *A. rivularis* and *P. viviparum*, which were either unchanged or significantly delayed in both 2017 and 2018 (**Figures 1B, D**, **Tables S2 and S3**). The advance of fruiting offset time was less conspicuous than other phenologies. It was significant for 5 out of 11 species in 2017 and 2 out of 8 species in 2018 (**Figures 2B, D**, **Tables S2 and S3**).

**Figure 3:**
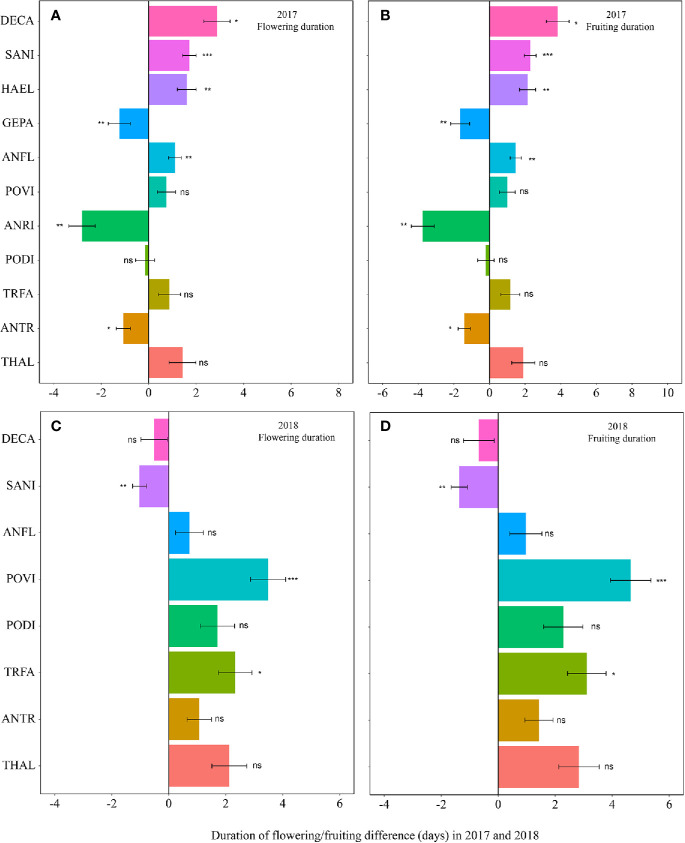
Changes in the flowering duration (**A**, 2017; **C**, 2018), and fruiting duration (**B**, 2017; **D**, 2018) (in days) between the non-warmed and warmed chambers from 2017 to 2018. Species are listed in the order from the earliest flowering species *Thalictrum alpinum* to the latest flowering species *Delphinium caeruleum*. A negative value indicates the duration of flowering or fruiting shortened in warmed chambers, while a positive value indicates the phenologies extended in warmed chambers. Bars indicate the mean ± SE for each bar. The difference in each phenology is determined by generalized linear mixed models (GLMMs). *P < 0.05; **P < 0.01; ***P < 0.001; ns, non-significant. The abbreviation of species names is same as **Figure 1**.

The warming effect was not statistically significant on the durations of flowering and fruiting for most plant species (**Table 1**, **Table S3**). Warming extended but not significantly the flowering and fruiting durations in both 2017 and 2018 (averagely 1.7 days for flowering duration and 2.2 days for fruiting duration) (**Figure 3**, **Table S3**). However, the magnitudes of the changes of the durations was both species- and year-specific.

### Relationships Between Plant Traits and Reproductive Phenophases

**Figure 4:**
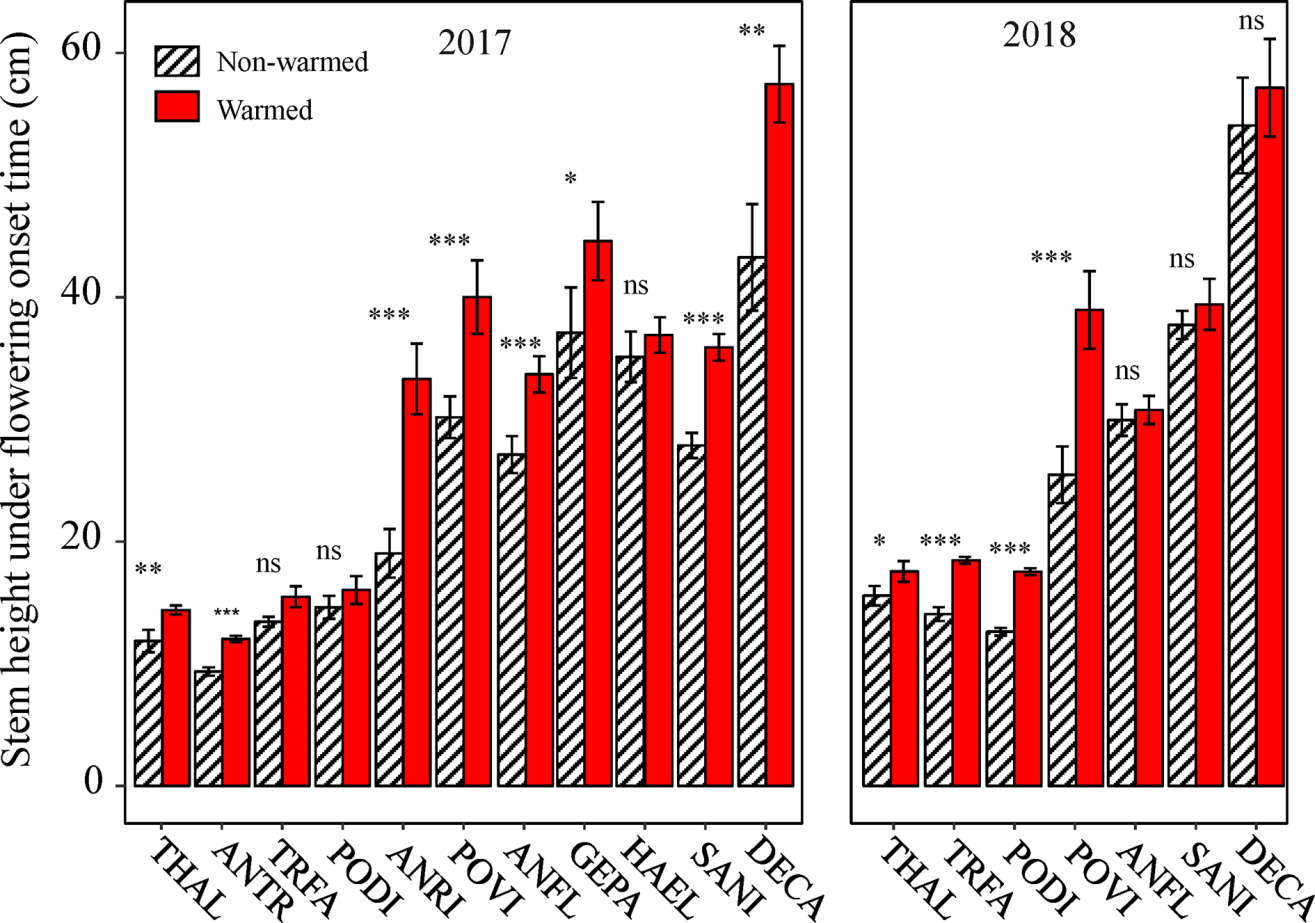
The effects of warming on plant height at flowering onset time for the study species from 2017 to 2018. Bars indicate the mean ± SE for each bar. Species are listed in the order form the earliest to latest flowering species. The difference in plant height is determined by generalized linear mixed models (GLMMs). *P < 0.05; **P < 0.01; ***P < 0.001; ns, non-significant. The abbreviation of species names is same as **Figure 1**.

**Figure 5:**
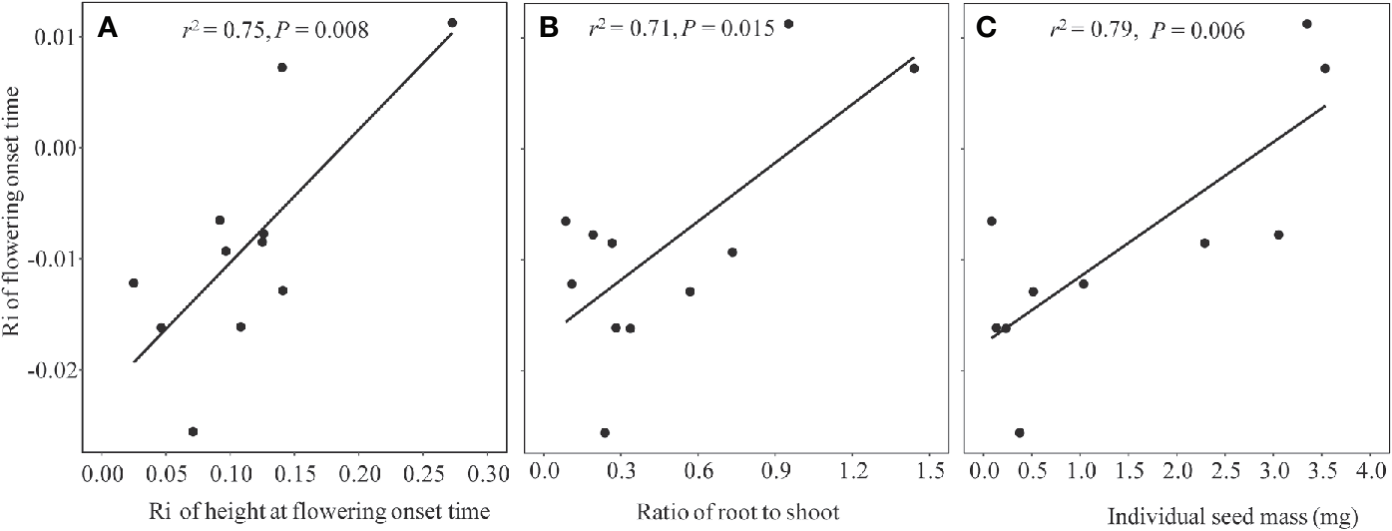
The regression relationship between Ri index of flowering onset time and functional traits in 2017, in which more species were investigated. **(A)** Ri index of plant height at the flowering onset time, **(B)** Biomass ratio of root to shoot; **(C)** Individual seed mass (mg).

Because all of the phenophases were highly correlated with each other in both non-warmed and warmed chambers (Pearson’s correlation: *r^2^* ≥ 0.94, *P* < 0.001 and *r^2^* ≥ 0.93, *P* < 0.001, respectively), only the relationship between flowering onset time and plant traits were explored. Plant height at flowering onset time was greater for most of the study species in warmed than in non-warmed chambers (**Figure 4**). The Ri of flowering onset time was positively correlated with the Ri of Hf (*r^2^* = 0.75, *P* = 0.008), and with R/S (*r^2^* = 0.71, *P* = 0.015; **Figures 5A, B**). Individual seed mass was positively correlated with the Ri of flowering onset time (*r^2^* = 0.79, *P* = 0.006; **Figure 5C**). However, the relationship between individual seed mass and the duration of fruit development was not significant (non-warmed: *r^2^* = −0.18, *P* = 0.62; warmed: *r^2^* = −0.35, *P* = 0.32).

## Discussion

The data presented here show that our experimental setting resulted in a mild winter-biased warming, with a slight increase in annual mean temperature and a higher increase in winter than in summer. This warming effect has not been commonly observed in previous studies (but Suonan et al., 2016) and stand in contrast to many other open top chambers (OTCs) studies, which often achieve a summer-biased warming. One explanation for the difference reported here and in other studies may be due to the fact that wind is usually much stronger in the winter compared to the summer. Results similar to those reported here have been reported in the same study region. The results reported here for a mild winter-biased warming are consistent with the prediction of IPCC (IPCC, 2007), and thus deserves further investigation. Winter-biased warming significantly changed plant flowering and fruiting phenologies, especially onset and offset times. Because flowering onset time is positively associated with flowering offset time and the onset and offset time of fruiting, flowering onset time will be focused for the following discussion.

Although this study shows that the phenological response to warming is species-specific, most of the species in this study advanced but not delayed their flowering, a phenomenology that is similar to the findings of other experimental warming studies performed in steppes, prairies, and alpine meadows (Arft et al., 1999; Sherry et al., 2007; Post et al., 2008; Dorji et al., 2013; Jabis et al., 2020). Moreover, other experimental warming studies in the northeast of Tibetan Plateau (Haibei Alpine Meadow) have shown an advance in the onset time of flowering. In particular, using infrared heaters simulating a winter warming, Suonan et al. (2016) reported that flowering onset time is advanced on average by 12.6 days in an alpine meadow, a duration considerably greater than that observed in our study (4–5 days at most). This difference may be attributed to the difference in the temperature increase reported by Suonan et al. (2016) and in our study (i.e., > 1.5°C and < 1°C, respectively).

The advance of flowering in our study indicates that winter warming expedites flower differentiation and development. Therefore, the vernalization hypothesis (i.e., warming decreases the strength of vernalization and delays plant leafing, flowering, and fruiting; see Körner and Basler, 2010) alone cannot explain our data. Instead, the advance in flowering onset time is more consistent with the accumulative temperature hypothesis (i.e., plants will flower when the accumulative temperatures reach a threshold; see Harrington and Gould, 2015). Nevertheless, it is possible that both hypotheses work as an explanation for the shift in flowering phenology such that the latter has an overriding influence, leading to an advance of flowering.

An additional observation is that plant height at the onset time of flowering is greater in warmed OTCs than in non-warmed OTCs, indicating that plants do not necessarily flower after reaching a specific height threshold. It is possible that plants only flower when they reach their maximum (physiologically optimal) height compared to other conspecifics (as suggested by the larger plant height at flowering onset time in the warmed chambers and the positive relationship between height at flowering onset time and flowering). If true, this supports the overtopping hypothesis. Achieving a maximum height may provide an advantage in attracting pollinators, because pollinators are generally scare (Peng et al., 2018). Because both the vernalization and accumulative temperature hypotheses are insufficient to explain a winter-biased warming-induced advance of flowering phenology, the overtopping hypothesis must be considered a potential candidate mechanism underlying the winter-biased warming-induced change in flowering phenology.

Although phenological advance under warming conditions is common across different ecosystems, exceptions have been observed (Price and Waser, 1998; Yu et al., 2010), i.e., not all species show the same direction and magnitude of changes in flowering time. The significant correlation between plant functional traits and changes in the onset time of flowering indicates that the response of flowering phenology to warming might be mediated by the former. One functional trait is growth in height. If warmed plants advance their shoot growth and flowering, they may avoid shading by neighbors, such that a small change in height at flowering onset time is sufficient to achieve “overtopping” success (Wesselingh et al., 1997; Jacquemyn et al., 2010). In contrast, if warmed plants delay their shoot growth and flowering, they must grow more in height to achieve competitive equality (i.e., a greater height at flowering onset time). This is implied by the positive relationship between the changes in flowering onset time and plant height at flowering onset time. The second functional trait is the R/S ratio. Plants with a lower R/S value are often more sensitive to lower soil moistures (see **Figure S7**), resulting in a slight drought that likely facilitates flowering (e.g. Yang et al., 2014). This is perhaps the reason why the species with lower R/S values advanced more in flowering than those with higher R/S. In addition, species with small seeds advance in flowering more than large-seeded species (**Figure 5C**). This positive relationship is unexpected but reasonable. Probably because the large-seeded species are taller at flowering onset time in warmed chambers (N = 10, *r^2^* = 0.33, *P* < 0.05), they could have enough energy to produce large seeds in short periods of time (thus allowing for a smaller advance or even delay in flowering onset time). However, no significant relationship between the seed size and fruiting period was found, inconsistent with the seed size-time hypothesis. This inconsistency may be attributed to grazing exclusion in the chambers. Plants are normally grazed by cattle and preventing grazing may change plant vegetative growth (Diaz et al., 2007), and reproductive phenologies (Li et al., 2019), thereby disrupting the size-time relationship.

Similar to the onset and offset times, the responses of the duration of flowering and fruiting are also diverse among the study species. It seems that the durations could be lengthened, unchanged, or shortened in both study year. This is consistent with the observation that the effects of climate change on the duration of reproduction are diverse (Price and Waser, 1998; Sherry et al., 2007; Post et al., 2008; Jiang et al., 2016). The mechanisms underlying the diverse response are unknown. Because flowering and fruiting durations are crucial to the performance of pollinators and herbivorous seed predators (Brody, 1997; Gervasi and Schiestl, 2017), the changed duration may induce change in higher trophic communities.

It is worthwhile to note that two common species *P. viviparum* and *A. rivularis* delayed their flowering. The delay can be explained by the vernalization hypothesis but not the accumulated temperature hypothesis. Moreover, *P. viviparum*, as a clonal species with bulbils, might have allocated its energy to asexual production first and then to sexual production in favorable environments. The flowering delay in the species *A. rivularis* can be explained by the change in flowering plant height, as indicated by the positive relationship between the changes in flowering height and flowering onset time.

## Conclusion

In summary, winter-biased warming significantly changed species reproductive phenologies with most species advancing their flowering, as has been observed in previous warming studies using infrared heaters and open top chambers. These changes are inconsistent with the vernalization hypothesis, but can be partly explained by the accumulated temperature hypothesis and/or the overtopping hypothesis. This observation suggests to us that both summer-biased and winter-biased warming may result in similar changes in phenology, or that phenological changes are subject to temperature increases regardless of the season. Our data also show that not all species have the same responses (or magnitude) of change, which is also widely recorded in previous studies. We have provided a preliminary mechanistic explanation for species-specific differences, i.e., different functional traits mediate different phenological responses to warming. Even a mild warming may trigger significant changes in plant phenology, and species-specific traits can affect different reproductive phenological responses in a future much warmed world.

## Data Availability Statement

Plant phenological and traits data for this study are included in Supplementary Material.

## Author Contributions

SS started the project, design research, and did major revision to the manuscript. XH and WZ performed research. XH analyzed data and drafted the manuscript.

## Funding

This study was financially supported by National Science Foundation of China (31530007 and 31325004).

## Conflict of Interest

The authors declare that the research was conducted in the absence of any commercial or financial relationships that could be construed as a potential conflict of interest.
