# Supplementary material for: Responses of Plant Reproductive Phenology to Winter-Biased Warming in an Alpine Meadow
Source: Front Plant Sci. 2020 Sep 4;11:534703. doi: 10.3389/fpls.2020.534703 (PMC7498618; doi:10.3389/fpls.2020.534703)

Supplementary Material

**Table S1** Statistical description for the warming effect on temperate differences at different levels of vertical profiles. Means of the temperature difference between warmed open top chambers (OTCs) and ambient OTCs were provided for both the growing and non-growing seasons. The data were complete except during the non-growing season from Apr. 2015 to Sep. 2019.

|  |  | 5cm underground (°C) | 30cm aboveground (°C) |
| --- | --- | --- | --- |
|  |  |  |  |
| Growing season  (Apr. 2015-Sep. 2015) | Range in warmed OTCs | 9.1-15.2 | 5.9-14.0 |
|  | Range in ambient OTCs | 9.0-15.7 | 4.7-13.9 |
|  | Mean of the difference | -0.33 | 0.28 |
| Non-growing season  (Oct. 2015-Mar. 2016) | Range in warmed OTCs | -0.5-9.3 | —— |
|  | Range in ambient OTCs | -3.1-11.3 | —— |
|  | Mean of the difference | 0.58 | —— |
| Growing season  (Apr. 2016-Sep. 2016) | Range in warmed OTCs | 7.8-15.6 | 6.5-15.7 |
|  | Range in ambient OTCs | 7.8-15.6 | 6.7-15.1 |
|  | Mean of the difference | -0.13 | 0.37 |
| Non-growing season  (Oct. 2016-Mar. 2017) | Range in warmed OTCs | —— | —— |
|  | Range in ambient OTCs | —— | —— |
|  | Mean of the difference | —— | —— |
| Growing season  (Apr. 2017-Sep. 2017) | Range in warmed OTCs | 7.3-14.2 | —— |
|  | Range in ambient OTCs | 7.7-15.1 | —— |
|  | Mean of the difference | -0.7 | —— |
| Non-growing season  (Oct. 2017-Mar. 2018) | Range in warmed OTCs | -0.5-9.3 | -4.3-7.8 |
|  | Range in ambient OTCs | -2.9-8.9 | -5-7.4 |
|  | Mean of the difference | 1.21 | 0.57 |
| Growing season (Apr. 2018-Sep. 2018) | Range in warmed OTCs | 6.4-16 | 7.1-15.6 |
|  | Range in ambient OTCs | 7.6-16.6 | 6.6-15.6 |
|  | Mean of the difference | -0.06 | 0.18 |
| Non-growing season (Oct. 2018-Mar. 2019) | Range in warmed OTCs | -0.7-8 | -6.3-3.6 |
|  | Range in ambient OTCs | -3.6-7.5 | -7.6-3.3 |
|  | Mean of the difference | 1.5 | 0.7 |
| Growing season (May 2019-Sep. 2019) | Range in warmed OTCs | 12.7-15.5 | 7.2-13.8 |
|  | Range in ambient OTCs | 13.5-15.9 | 6.9-13.8 |
|  | Mean of the difference | -0.9 | 0.1 |
| Annual mean (Oct. 2015-Sep. 2016) | Range in warmed OTCs | -3.1-15.5 | —— |
|  | Range in ambient OTCs | -4.4-15.6 | —— |
|  | Mean of the difference | 0.98 | —— |
| Annual mean (Oct. 2016-Sep. 2017) | Range in warmed OTCs | —— | —— |
|  | Range in ambient OTCs | —— | —— |
|  | Mean of the difference | —— | —— |
| Annual mean (Oct. 2017-Sep. 2018) | Range in warmed OTCs | -0.45-16.0 | -5-15.6 |
|  | Range in ambient OTCs | -2.9-16.6 | -4.3-15.6 |
|  | Mean of the difference | 0.54 | 0.37 |
| Annual mean (Oct. 2018-Sep. 2019) | Range in warmed OTCs | -3.6-15.9 | -6.3-13.8 |
|  | Range in ambient OTCs | -0.7-15.5 | -7.6-13.8 |
|  | Mean of the difference | 0.31 | 0.4 |

**Table S2** The means ± SE of the flowering and fruiting onset /offset time, and duration of flowering and fruiting for all the study species in both warmed and un-warmed treatments in 2017 and 2018. The difference in each phenology is determined by generalized linear mixed models (GLMMs). *P* values are provided and those with significant differences are bold.

| **Year** | **Phenophase** | **Species** | **Non-warmed (Julian day)** | **Warmed (Julian day)** | ***P* value** |
| --- | --- | --- | --- | --- | --- |
| 2017 | Flowering onset | *Thalictrum alpinum* | 127.4 ± 0.98 | 125 ± 1.55 | 0.189 |
|  |  | *Anemone trullifolia var. linearis* | 142.5 ± 0.67 | 140.1 ± 0.54 | **0.008** |
|  |  | *Trollius farreri* | 151.1 ± 1.02 | 143.6 ± 1.11 | **0.000** |
|  |  | *Potentilla discolor* | 163.9 ± 0.89 | 158.7 ± 1.11 | **0.000** |
|  |  | *Anemone rivularis* | 170 ± 1.41 | 173.9 ± 1.25 | **0.002** |
|  |  | *Polygonum viviparum* | 178 ± 0.85 | 180.6 ± 0.7 | **0.036** |
|  |  | *Anaphalis flavescens* | 185.3 ± 0.64 | 179.4 ± 0.42 | **0.000** |
|  |  | *Gentianopsis paludosa* | 186.2 ± 0.55 | 183.8 ± 0.64 | **0.004** |
|  |  | *Halenia elliptica* | 195.3 ± 0.95 | 190.6 ± 1.03 | **0.002** |
|  |  | *Saussurea nigrescens* | 201.5 ± 0.74 | 198.4 ± 0.47 | **0.001** |
|  |  | *Delphinium caeruleum* | 206.6 ± 1.73 | 201.3 ± 1.07 | **0.010** |
|  | Flowering offset | *Thalictrum alpinum* | 151.9 ± 0.59 | 151 ± 0.7 | 0.335 |
|  |  | *Anemone trullifolia var. linearis* | 162.5 ± 0.67 | 159 ± 0.34 | **0.000** |
|  |  | *Trollius farreri* | 172.8 ± 1.22 | 166.1 ± 0.99 | **0.000** |
|  |  | *Potentilla discolor* | 184.3 ± 0.71 | 178.9 ± 0.85 | **0.000** |
|  |  | *Anemone rivularis* | 190.5 ± 1.14 | 191.6 ± 0.69 | 0.413 |
|  |  | *Polygonum viviparum* | 189.8 ± 0.98 | 193.2 ± 1.14 | **0.022** |
|  |  | *Anaphalis flavescens* | 202.3 ± 0.62 | 197.5 ± 0.39 | 0.062 |
|  |  | *Gentianopsis paludosa* | 201.8 ± 0.85 | 198.1 ± 0.63 | **0.001** |
|  |  | *Halenia elliptica* | 207.7 ± 0.48 | 204.6 ± 0.71 | **0.002** |
|  |  | *Saussurea nigrescens* | 216.9 ± 0.53 | 215.5 ± 0.52 | 0.062 |
|  |  | *Delphinium caeruleum* | 222.3 ± 1.04 | 220 ± 0.69 | 0.081 |
|  | Fruiting onset | *Thalictrum alpinum* | 160 ± 0.59 | 159.6 ± 0.7 | 0.623 |
|  |  | *Anemone trullifolia var. linearis* | 169.2 ± 0.67 | 165.4 ± 0.34 | **0.000** |
|  |  | *Trollius farreri* | 180 ± 1.22 | 173.6 ± 0.99 | **0.000** |
|  |  | *Potentilla discolor* | 191.1 ± 0.71 | 185.7 ± 0.85 | **0.000** |
|  |  | *Anemone rivularis* | 197.4 ± 1.14 | 197.5 ± 0.69 | **0.923** |
|  |  | *Polygonum viviparum* | 193.8 ± 0.98 | 197.4 ± 1.14 | **0.023** |
|  |  | *Anaphalis flavescens* | 207.9 ± 0.62 | 203.5 ± 0.39 | **0.000** |
|  |  | *Gentianopsis paludosa* | 207 ± 0.85 | 202.9 ± 0.63 | **0.000** |
|  |  | *Halenia elliptica* | 211.9 ± 0.48 | 209.3 ± 0.71 | **0.005** |
|  |  | *Saussurea nigrescens* | 222 ± 0.53 | 221.2 ± 0.52 | 0.279 |
|  |  | *Delphinium caeruleum* | 227.6 ± 1.04 | 226.2 ± 0.69 | 0.262 |
|  | Fruiting offset | *Thalictrum alpinum* | 192.7 ± 0.94 | 194.1 ± 1.85 | 0.473 |
|  |  | *Anemone trullifolia var. linearis* | 195.9 ± 1 | 190.6 ± 0.52 | **0.000** |
|  |  | *Trollius farreri* | 208.8 ± 1.86 | 203.6 ± 1.39 | **0.023** |
|  |  | *Potentilla discolor* | 218.3 ± 0.98 | 212.6 ± 1.08 | **0.000** |
|  |  | *Anemone rivularis* | 224.8 ± 2.17 | 221.2 ± 0.52 | 0.710 |
|  |  | *Polygonum viviparum* | 209.6 ± 1.33 | 214.2 ± 1.74 | **0.037** |
|  |  | *Anaphalis flavescens* | 230.5 ± 0.9 | 227.6 ± 0.56 | **0.004** |
|  |  | *Gentianopsis paludosa* | 227.7 ± 1.44 | 222 ± 0.87 | **0.000** |
|  |  | *Halenia elliptica* | 228.4 ± 0.52 | 228 ± 0.6 | 0.710 |
|  |  | *Saussurea nigrescens* | 242.6 ± 0.63 | 244.1 ± 0.87 | 0.159 |
|  |  | *Delphinium caeruleum* | 248.6 ± 1.18 | 251.1 ± 1.37 | 0.188 |
|  | Flowering duration | *Thalictrum alpinum* | 24.5 ± 0.57 | 25.9 ± 1.17 | 0.255 |
|  |  | *Anemone trullifolia var. linearis* | 20 ± 0.4 | 19 ± 0.31 | **0.028** |
|  |  | *Trollius farreri* | 21.6 ± 0.65 | 22.5 ± 0.58 | 0.309 |
|  |  | *Potentilla discolor* | 20.4 ± 0.46 | 20.2 ± 0.52 | 0.819 |
|  |  | *Anemone rivularis* | 20.5 ± 1.07 | 17.7 ± 0.5 | **0.007** |
|  |  | *Polygonum viviparum* | 11.9 ± 0.41 | 12.6 ± 0.51 | 0.258 |
|  |  | *Anaphalis flavescens* | 16.9 ± 0.35 | 18 ± 0.23 | **0.009** |
|  |  | *Gentianopsis paludosa* | 15.6 ± 0.52 | 14.3 ± 0.32 | **0.006** |
|  |  | *Halenia elliptica* | 12.4 ± 0.45 | 14 ± 0.35 | **0.007** |
|  |  | *Saussurea nigrescens* | 15.4 ± 0.33 | 17.1 ± 0.35 | **0.000** |
|  |  | *Delphinium caeruleum* | 15.8 ± 0.79 | 18.7 ± 0.76 | **0.010** |
|  | Fruiting duration | *Thalictrum alpinum* | 32.7 ± 0.76 | 34.6 ± 1.56 | 0.255 |
|  |  | *Anemone trullifolia var. linearis* | 26.7 ± 0.53 | 25.3 ± 0.41 | **0.028** |
|  |  | *Trollius farreri* | 28.8 ± 0.87 | 30 ± 0.77 | 0.309 |
|  |  | *Potentilla discolor* | 27.2 ± 0.62 | 27 ± 0.69 | 0.819 |
|  |  | *Anemone rivularis* | 27.4 ± 1.43 | 23.6 ± 0.66 | **0.007** |
|  |  | *Polygonum viviparum* | 15.8 ± 0.54 | 16.8 ± 0.68 | 0.258 |
|  |  | *Anaphalis flavescens* | 22.6 ± 0.47 | 24.1 ± 0.31 | **0.009** |
|  |  | *Gentianopsis paludosa* | 20.8 ± 0.69 | 19.1 ± 0.42 | **0.006** |
|  |  | *Halenia elliptica* | 16.5 ± 0.59 | 18.7 ± 0.47 | **0.007** |
|  |  | *Saussurea nigrescens* | 20.6 ± 0.44 | 22.9 ± 0.47 | **0.000** |
|  |  | *Delphinium caeruleum* | 21 ± 1.06 | 24.9 ± 1.01 | **0.010** |
| 2018 | Flowering onset | *Thalictrum alpinum* | 125.2 ± 1.42 | 120.4 ± 2.11 | 0.056 |
|  |  | *Anemone trullifolia var. linearis* | 145.3 ± 0.26 | 143.9 ± 0.68 | **0.041** |
|  |  | *Trollius farreri* | 149.8 ± 1.58 | 141.8 ± 1.31 | **0.000** |
|  |  | *Potentilla discolor* | 158.4 ± 1.08 | 151.6 ± 1.46 | **0.000** |
|  |  | *Polygonum viviparum* | 165.6 ± 0.88 | 173.9 ± 1.49 | **0.000** |
|  |  | *Anaphalis flavescens* | 176.6 ± 0.84 | 172.6 ± 0.76 | **0.001** |
|  |  | *Saussurea nigrescens* | 192.9 ± 0.44 | 189.1 ± 0.53 | **0.000** |
|  |  | *Delphinium caeruleum* | 208.4 ± 1.08 | 204.5 ± 0.79 | **0.003** |
|  | Flowering offset | *Thalictrum alpinum* | 153.5 ± 1.41 | 150.8 ± 2.61 | 0.091 |
|  |  | *Anemone trullifolia var. linearis* | 159.1 ± 1.91 | 158.8 ± 1.73 | 0.726 |
|  |  | *Trollius farreri* | 169.4 ± 0.77 | 163.7 ± 0.57 | **0.002** |
|  |  | *Potentilla discolor* | 179.6 ± 0.52 | 174.5 ± 0.58 | **0.004** |
|  |  | *Polygonum viviparum* | 176.8 ± 2.24 | 188.6 ± 2.29 | **0.000** |
|  |  | *Anaphalis flavescens* | 202.1 ± 0.67 | 198.8 ± 0.93 | **0.000** |
|  |  | *Saussurea nigrescens* | 213.8 ± 1.1 | 208.9 ± 1.2 | **0.000** |
|  |  | *Delphinium caeruleum* | 222.1 ± 1 | 217.7 ± 1.19 | **0.000** |
|  | Fruiting onset | *Thalictrum alpinum* | 163 ± 1.1 | 161 ± 1.2 | 0.185 |
|  |  | *Anemone trullifolia var. linearis* | 163.7 ± 0.52 | 163.7 ± 0.58 | 0.857 |
|  |  | *Trollius farreri* | 175.9 ± 1.4 | 171 ± 1.11 | **0.009** |
|  |  | *Potentilla discolor* | 186.7 ± 1.06 | 182.1 ± 1.6 | **0.019** |
|  |  | *Polygonum viviparum* | 180.6 ± 1.41 | 193.5 ± 2.61 | **0.000** |
|  |  | *Anaphalis flavescens* | 210.6 ± 1 | 207.6 ± 1.19 | **0.053** |
|  |  | *Saussurea nigrescens* | 220.8 ± 0.46 | 215.6 ± 0.63 | **0.000** |
|  |  | *Delphinium caeruleum* | 226.7 ± 0.77 | 222.1 ± 0.57 | **0.000** |
|  | Fruit offset | *Thalictrum alpinum* | 200.7 ± 1.97 | 201.6 ± 1.46 | 0.731 |
|  |  | *Anemone trullifolia var. linearis* | 182.1 ± 1.04 | 183.6 ± 1.08 | 0.305 |
|  |  | *Trollius farreri* | 202 ± 1.91 | 200.2 ± 1.73 | 0.500 |
|  |  | *Potentilla discolor* | 214.9 ± 2.24 | 212.6 ± 2.29 | 0.489 |
|  |  | *Polygonum viviparum* | 195.5 ± 2.11 | 213.2 ± 4.11 | **0.000** |
|  |  | *Anaphalis flavescens* | 244.6 ± 1.89 | 242.6 ± 2.26 | 0.494 |
|  |  | *Saussurea nigrescens* | 248.6 ± 0.67 | 242 ± 0.93 | **0.000** |
|  |  | *Delphinium caeruleum* | 245 ± 0.95 | 239.7 ± 0.86 | **0.000** |
|  | Flowering duration | *Thalictrum alpinum* | 28.3 ± 0.99 | 30.4 ± 1.02 | 0.138 |
|  |  | *Anemone trullifolia var. linearis* | 13.8 ± 0.41 | 14.9 ± 0.52 | 0.103 |
|  |  | *Trollius farreri* | 19.6 ± 0.79 | 21.9 ± 0.79 | **0.048** |
|  |  | *Potentilla discolor* | 21.2 ± 1.05 | 22.9 ± 0.79 | 0.227 |
|  |  | *Polygonum viviparum* | 11.2 ± 0.59 | 14.7 ± 1.25 | **0.000** |
|  |  | *Anaphalis flavescens* | 25.5 ± 0.79 | 26.2 ± 0.9 | 0.545 |
|  |  | *Saussurea nigrescens* | 20.9 ± 0.25 | 19.8 ± 0.31 | **0.005** |
|  |  | *Delphinium caeruleum* | 13.7 ± 0.5 | 13.2 ± 0.45 | 0.588 |
|  | Fruiting duration | *Thalictrum alpinum* | 37.8 ± 1.32 | 40.6 ± 1.36 | 0.138 |
|  |  | *Anemone trullifolia var. linearis* | 18.4 ± 0.55 | 19.8 ± 0.7 | 0.103 |
|  |  | *Trollius farreri* | 26.1 ± 1.05 | 29.2 ± 1.06 | **0.048** |
|  |  | *Potentilla discolor* | 28.2 ± 1.4 | 30.5 ± 1.05 | 0.227 |
|  |  | *Polygonum viviparum* | 15 ± 0.79 | 19.6 ± 1.67 | **0.000** |
|  |  | *Anaphalis flavescens* | 34 ± 1.06 | 35 ± 1.2 | 0.545 |
|  |  | *Saussurea nigrescens* | 27.8 ± 0.33 | 26.5 ± 0.41 | **0.005** |
|  |  | *Delphinium caeruleum* | 18.3 ± 0.66 | 17.6 ± 0.6 | 0.588 |

**Table S3** Summary of the GLMMs analysis of variance of the six phenological events (onset/offset time, duration of flowering and fruiting) for each species. The “year” of eight species (*Thalictrum alpinum*; *Anemone trullifolia var. linearis*; *Trollius farreri*; *Potentilla discolor*; *Polygonum viviparum*; *Anaphalis flavescens*; *Saussurea nigrescens* and *Delphinium caeruleum*) observed in consecutive years were included. *, *P* < 0.05; **, *P* < 0.01; ***, *P* < 0.001.

|  | ***Thalictrum alpinum*** | | | | |  | ***Anemone trullifolia var. linearis*** | | | |
| --- | --- | --- | --- | --- | --- | --- | --- | --- | --- | --- |
| **Flowering onset time** | **Source** | **NumDF** | **DenDF** | ***F*** | ***P*** |  | **NumDF** | **DenDF** | ***F*** | ***P*** |
|  | **Warming (W)** | 1 | 103.38 | 5.7844 | 0.01795* |  | 1 | 180 | 6.2466 | 0.01334* |
|  | **Year (Y)** | 1 | 103.22 | 5.1585 | 0.02521* |  | 1 | 180 | 19.3268 | <0.0001*** |
|  | **W : Y** | 1 | 103.38 | 0.7343 | 0.39347 |  | 1 | 180 | 0.4797 | 0.48943 |
|  |  |  |  |  |  |  |  |  |  |  |
| **Flowering offset time** | **Warming (W)** | 1 | 103.41 | 4.0469 | 0.04685* |  | 1 | 151.91 | 8.6947 | 0.003696** |
|  | **Year (Y)** | 1 | 103.16 | 0.648 | 0.42269 |  | 1 | 158.13 | 7.611 | 0.006486** |
|  | **W : Y** | 1 | 103.41 | 1.0337 | 0.31167 |  | 1 | 150.48 | 6.5013 | 0.01178* |
|  |  |  |  |  |  |  |  |  |  |  |
| **Fruiting onset time** | **Warming (W)** | 1 | 103.6 | 1.9952 | 0.1608 |  | 1 | 152.38 | 8.1448 | 0.00492** |
|  | **Year (Y)** | 1 | 103.31 | 5.8356 | 0.01746* |  | 1 | 152.34 | 25.4947 | <0.0001*** |
|  | **W : Y** | 1 | 103.6 | 0.8277 | 0.36505 |  | 1 | 151.41 | 9.3423 | 0.002648** |
|  |  |  |  |  |  |  |  |  |  |  |
| **Fruiting offset time** | **Warming (W)** | 1 | 113 | 0.519 | 0.4728 |  | 1 | 153.77 | 3.4976 | 0.0633574 |
|  | **Year (Y)** | 1 | 113 | 23.625 | <0.0001*** |  | 1 | 149.31 | 94.8004 | <0.0001*** |
|  | **W : Y** | 1 | 113 | 0.0369 | 0.8481 |  | 1 | 153.19 | 12.7 | 0.0004879*** |
|  |  |  |  |  |  |  |  |  |  |  |
| **Floweing duration** | **Warming (W)** | 1 | 104.23 | 3.4318 | 0.06678 |  | 1 | 156.16 | 0.0024 | 0.96079 |
|  | **Year (Y)** | 1 | 104.55 | 18.9779 | <0.0001*** |  | 1 | 149.2 | 118.8486 | <0.0001*** |
|  | **W : Y** | 1 | 104.23 | 0.1339 | 0.7152 |  | 1 | 155.47 | 6.2245 | 0.01364* |
|  |  |  |  |  |  |  |  |  |  |  |
| **Fruiting duration** | **Warming (W)** | 1 | 104.22 | 3.4318 | 0.06678 |  | 1 | 156.16 | 0.0024 | 0.96079 |
|  | **Year (Y)** | 1 | 104.54 | 18.9778 | <0.0001*** |  | 1 | 149.2 | 118.8486 | <0.0001*** |
|  | **W : Y** | 1 | 104.22 | 0.1339 | 0.7152 |  | 1 | 155.47 | 6.2245 | 0.01364* |

|  | ***Trollius farreri*** | | | | |  | ***Potentilla discolor*** | | | |
| --- | --- | --- | --- | --- | --- | --- | --- | --- | --- | --- |
| **Flowering onset time** | **Source** | **NumDF** | **DenDF** | ***F*** | ***P*** |  | **NumDF** | **DenDF** | ***F*** | ***P*** |
|  | **Warming (W)** | 1 | 100.237 | 37.8518 | <0.0001*** | | 1 | 114.52 | 31.3254 | <0.0001*** |
|  | **Year (Y)** | 1 | 111.757 | 1.334 | 0.2506 |  | 1 | 114.81 | 33.8844 | <0.0001*** |
|  | **W : Y** | 1 | 99.185 | 0.0396 | 0.8427 |  | 1 | 114.94 | 0.4077 | 0.5244 |
|  |  |  |  |  |  |  |  |  |  |  |
| **Flowering offset time** | **Warming (W)** | 1 | 113 | 28.8752 | <0.0001*** | | 1 | 114.67 | 29.0889 | <0.0001*** |
|  | **Year (Y)** | 1 | 113 | 6.512 | 0.01205* |  | 1 | 120.37 | 21.8271 | <0.0001*** |
|  | **W : Y** | 1 | 113 | 0.1891 | 0.66452 |  | 1 | 114.55 | 0.0209 | 0.8854 |
|  |  |  |  |  |  |  |  |  |  |  |
| **Fruiting onset time** | **Warming (W)** | 1 | 113 | 21.982 | <0.0001*** | | 1 | 127 | 23.2566 | <0.0001*** |
|  | **Year (Y)** | 1 | 113 | 7.8706 | 0.005918** |  | 1 | 127 | 14.8594 | 0.000183*** |
|  | **W : Y** | 1 | 113 | 0.3814 | 0.53809 |  | 1 | 127 | 0.1909 | 0.662947 |
|  |  |  |  |  |  |  |  |  |  |  |
| **Fruiting offset time** | **Warming (W)** | 1 | 113 | 3.9308 | 0.04984* |  | 1 | 127 | 5.8861 | 0.01667* |
|  | **Year (Y)** | 1 | 113 | 8.5107 | 0.00426** |  | 1 | 127 | 1.093 | 0.29779 |
|  | **W : Y** | 1 | 113 | 0.9474 | 0.33245 |  | 1 | 127 | 1.0899 | 0.29847 |
|  |  |  |  |  |  |  |  |  |  |  |
| **Floweing duration** | **Warming (W)** | 1 | 101.121 | 5.0144 | 0.02733* |  | 1 | 115.61 | 1.2293 | 0.26985 |
|  | **Year (Y)** | 1 | 111.641 | 3.5069 | 0.06373 |  | 1 | 115.42 | 6.0396 | 0.01547* |
|  | **W : Y** | 1 | 99.308 | 1.0513 | 0.3077 |  | 1 | 116.37 | 1.619 | 0.20577 |
|  |  |  |  |  |  |  |  |  |  |  |
| **Fruiting duration** | **Warming (W)** | 1 | 101.121 | 5.0144 | 0.02733* |  | 1 | 115.61 | 1.2293 | 0.26985 |
|  | **Year (Y)** | 1 | 111.641 | 3.5069 | 0.06373 |  | 1 | 115.42 | 6.0396 | 0.01547* |
|  | **W : Y** | 1 | 99.308 | 1.0513 | 0.3077 |  | 1 | 116.37 | 1.619 | 0.20577 |

|  |  | ***Polygonum viviparum*** | |  |  |  | ***Anaphalis flavescens*** | | | |
| --- | --- | --- | --- | --- | --- | --- | --- | --- | --- | --- |
| **Flowering onset time** | **Source** | **NumDF** | **DenDF** | ***F*** | ***P*** |  | **NumDF** | **DenDF** | ***F*** | ***P*** |
|  | **Warming (W)** | 1 | 124 | 28.4873 | <0.0001*** | | 1 | 220.98 | 56.1083 | <0.0001*** |
|  | **Year (Y)** | 1 | 124 | 86.3903 | <0.0001*** | | 1 | 223.25 | 139.0602 | <0.0001*** |
|  | **W : Y** | 1 | 124 | 7.8485 | 0.005903** |  | 1 | 220.85 | 2.0886 | 0.1498 |
|  |  |  |  |  |  |  |  |  |  |  |
| **Flowering offset time** | **Warming (W)** | 1 | 113.15 | 34.573 | <0.0001*** | | 1 | 205.63 | 35.6615 | <0.0001*** |
|  | **Year (Y)** | 1 | 116.29 | 46.64 | <0.0001*** | | 1 | 208.33 | 0.7092 | 0.4007 |
|  | **W : Y** | 1 | 114.13 | 11.245 | 0.001084** |  | 1 | 205.72 | 1.4294 | 0.2332 |
|  |  |  |  |  |  |  |  |  |  |  |
| **Fruiting onset time** | **Warming (W)** | 1 | 112.84 | 33.813 | <0.0001*** | | 1 | 205.76 | 22.7595 | <0.0001*** |
|  | **Year (Y)** | 1 | 114.87 | 36.559 | <0.0001*** | | 1 | 208.83 | 18.3871 | <0.0001*** |
|  | **W : Y** | 1 | 113.83 | 11.429 | 0.0009915*** | | 1 | 205.71 | 0.9327 | 0.3353 |
|  |  |  |  |  |  |  |  |  |  |  |
| **Fruiting offset time** | **Warming (W)** | 1 | 112.07 | 28.532 | <0.0001*** | | 1 | 206.23 | 3.1984 | 0.07518 |
|  | **Year (Y)** | 1 | 112.11 | 14.483 | 0.000231*** | | 1 | 211.48 | 106.6046 | <0.0001*** |
|  | **W : Y** | 1 | 112.96 | 10.65 | 0.001456** |  | 1 | 205.86 | 0.1254 | 0.72361 |
|  |  |  |  |  |  |  |  |  |  |  |
| **Floweing duration** | **Warming (W)** | 1 | 110.85 | 11.9491 | 0.0007762*** | | 1 | 207.01 | 2.5545 | 0.1115 |
|  | **Year (Y)** | 1 | 109.22 | 0.8651 | 0.3543538 |  | 1 | 215.75 | 211.4831 | <0.0001*** |
|  | **W : Y** | 1 | 111.5 | 5.8236 | 0.0174426* |  | 1 | 206.39 | 0.12 | 0.7294 |
|  |  |  |  |  |  |  |  |  |  |  |
| **Fruiting duration** | **Warming (W)** | 1 | 110.85 | 11.9491 | 0.0007762*** | | 1 | 207.01 | 2.5545 | 0.1115 |
|  | **Year (Y)** | 1 | 109.22 | 0.8651 | 0.3543538 |  | 1 | 215.75 | 211.4831 | <0.0001*** |
|  | **W : Y** | 1 | 111.5 | 5.8236 | 0.0174426* |  | 1 | 206.39 | 0.12 | 0.7294 |

| ***Saussurea nigrescens*** | | | | | |  | ***Delphinium caeruleum*** | | | |
| --- | --- | --- | --- | --- | --- | --- | --- | --- | --- | --- |
| **Flowering onset time** | **Source** | **NumDF** | **DenDF** | ***F*** | ***P*** |  | ***NumDF*** | ***DenDF*** | ***F*** | ***P*** |
|  | **Warming (W)** | 1 | 272.79 | 40.5025 | <0.0001*** | | 1 | 80.092 | 12.1905 | 0.0007845*** |
|  | **Year (Y)** | 1 | 284.56 | 268.1647 | <0.0001*** | | 1 | 80.7 | 3.6993 | 0.0579609 |
|  | **W : Y** | 1 | 270.99 | 0.4269 | 0.5141 |  | 1 | 81.038 | 0.2853 | 0.5946998 |
|  |  |  |  |  |  |  |  |  |  |  |
| **Flowering offset time** | **Warming (W)** | 1 | 271.5 | 37.285 | <0.0001*** | | 1 | 81.046 | 14.4855 | 0.0002731*** |
|  | **Year (Y)** | 1 | 272.71 | 89.866 | <0.0001*** | | 1 | 81.438 | 2.0394 | 0.1570934 |
|  | **W : Y** | 1 | 270.19 | 11.62 | 0.0007522*** | | 1 | 81.844 | 1.3296 | 0.2522349 |
|  |  |  |  |  |  |  |  |  |  |  |
| **Fruiting onset time** | **Warming (W)** | 1 | 247.66 | 31.441 | <0.0001*** | | 1 | 81.838 | 12.8443 | 0.0005738*** |
|  | **Year (Y)** | 1 | 265.49 | 42.163 | <0.0001*** | | 1 | 82.144 | 9.1091 | 0.0033865** |
|  | **W : Y** | 1 | 245.8 | 16.799 | <0.0001*** | | 1 | 82.2 | 3.6155 | 0.0607463. |
|  |  |  |  |  |  |  |  |  |  |  |
| **Fruiting offset time** | **Warming (W)** | 1 | 238.13 | 11.4573 | 0.0008327*** | | 1 | 81.596 | 1.3983 | 0.240447 |
|  | **Year (Y)** | 1 | 259.99 | 7.6836 | 0.0059754** | | 1 | 82.114 | 40.0567 | <0.0001*** |
|  | **W : Y** | 1 | 236 | 27.543 | <0.0001*** | | 1 | 81.12 | 10.4746 | 0.001753** |
|  |  |  |  |  |  |  |  |  |  |  |
| **Floweing duration** | **Warming (W)** | 1 | 269.31 | 1.2176 | 0.2708 |  | 1 | 80.281 | 2.8887 | 0.09307 |
|  | **Year (Y)** | 1 | 286.58 | 202.4257 | <0.0001*** | | 1 | 80.75 | 29.4313 | <0.0001*** |
|  | **W : Y** | 1 | 268.51 | 22.2412 | <0.0001*** | | 1 | 80.337 | 6.192 | 0.0149* |
|  |  |  |  |  |  |  |  |  |  |  |
| **Fruiting duration** | **Warming (W)** | 1 | 269.31 | 1.2176 | 0.2708 |  | 1 | 80.281 | 2.8887 | 0.09307 |
|  | **Year (Y)** | 1 | 286.58 | 202.4257 | <0.0001*** | | 1 | 80.75 | 29.4313 | <0.0001*** |
|  | **W : Y** | 1 | 268.51 | 22.2412 | <0.0001*** | | 1 | 80.337 | 6.192 | 0.0149* |

|  | ***Anemone rivularis*** | | | | |  | ***Gentianopsis Paludosa*** | | | |  | ***Halenia elliptica*** | | | |
| --- | --- | --- | --- | --- | --- | --- | --- | --- | --- | --- | --- | --- | --- | --- | --- |
|  | **Source** | **NumDF** | **DenDF** | ***F*** | ***P*** |  | **NumDF** | **DenDF** | ***F*** | ***P*** |  | **NumDF** | **DenDF** | ***F*** | ***P*** |
| **Flowering onset time** | **Warming** | 1 | 44 | 4.216 | 0.04602* |  | 1 | 20.337 | 7.6432 | 0.01184* |  | 1 | 51 | 11.23 | 0.001522** |
| **Flowering offset time** | **Warming** | 1 | 37.649 | 0.6848 | 0.4132 |  | 1 | 19.872 | 15.04 | 0.0009445*** |  | 1 | 51 | 10.352 | 0.002249** |
| **Fruiting onset time** | **Warming** | 1 | 37.157 | 0.0094 | 0.9231 |  | 1 | 20.145 | 16.473 | 0.0006059*** |  | 1 | 50.998 | 9.0768 | 0.004023** |
| **Fruiting offset time** | **Warming** | 1 | 37.695 | 3.7159 | 0.06146 |  | 1 | 20.419 | 16.166 | 0.0006476*** |  | 1 | 42.038 | 0.1762 | 0.6768 |
| **Flowering duration** | **Warming** | 1 | 38.647 | 6.7139 | 0.01343* |  | 1 | 20.366 | 6.3464 | 0.02021* |  | 1 | 51 | 7.9449 | 0.006848** |
| **Fruiting duration** | **Warming** | 1 | 38.647 | 6.7139 | 0.01343* |  | 1 | 20.366 | 6.3464 | 0.02021* |  | 1 | 51 | 7.9449 | 0.006848** |

**Figure S1** The study site region and the experimental open top chambers. (A) The study site region, (B) The construction design of the open top chambers, (C) General physical view of open top chambers in construction, (D) The layout of the experimental open top chambers.

(A)


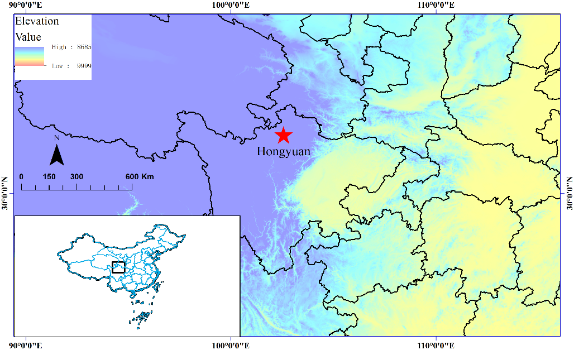


(B)


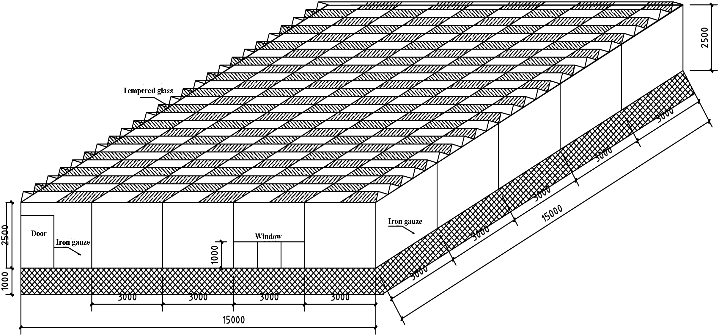


(C)


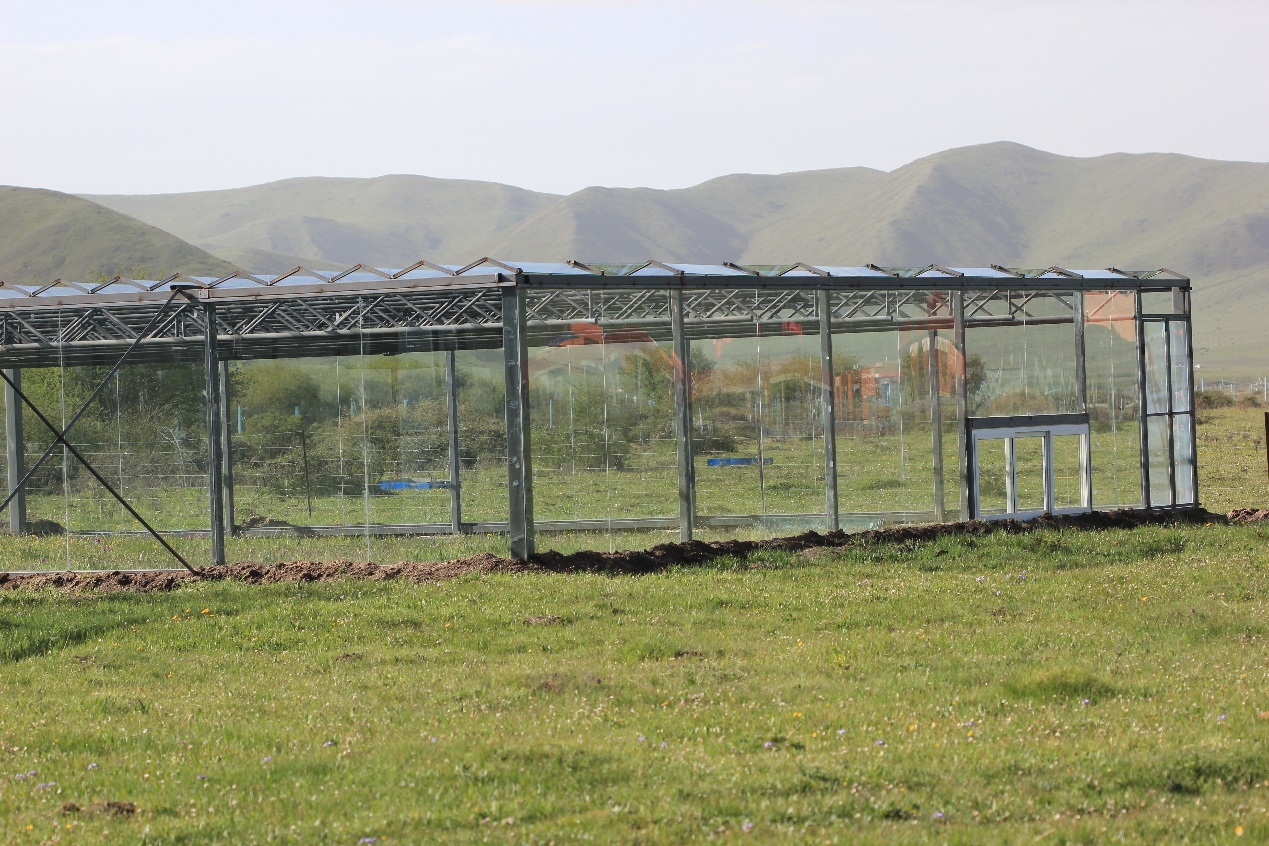


(D)


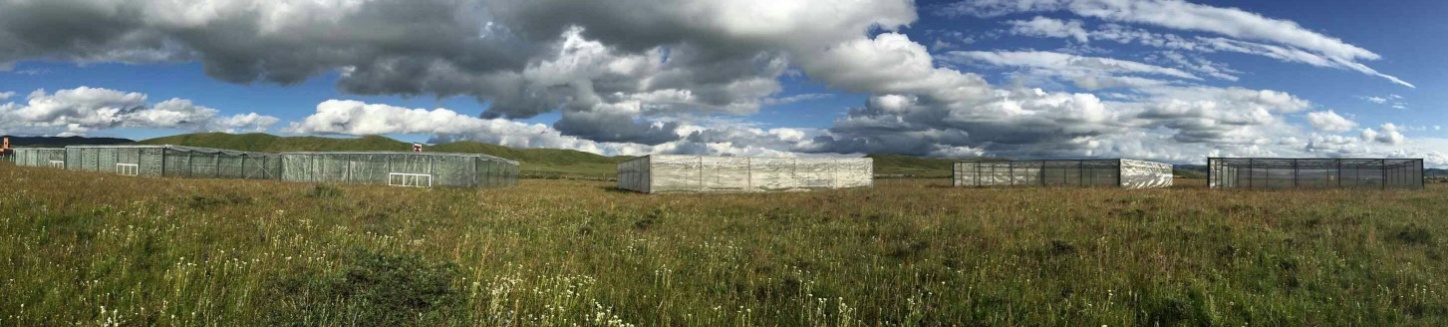


**Figure S2** Variation in temperature at 30cm above ground in the non-warmed and warmed treatments from September 2017 to November 2019.


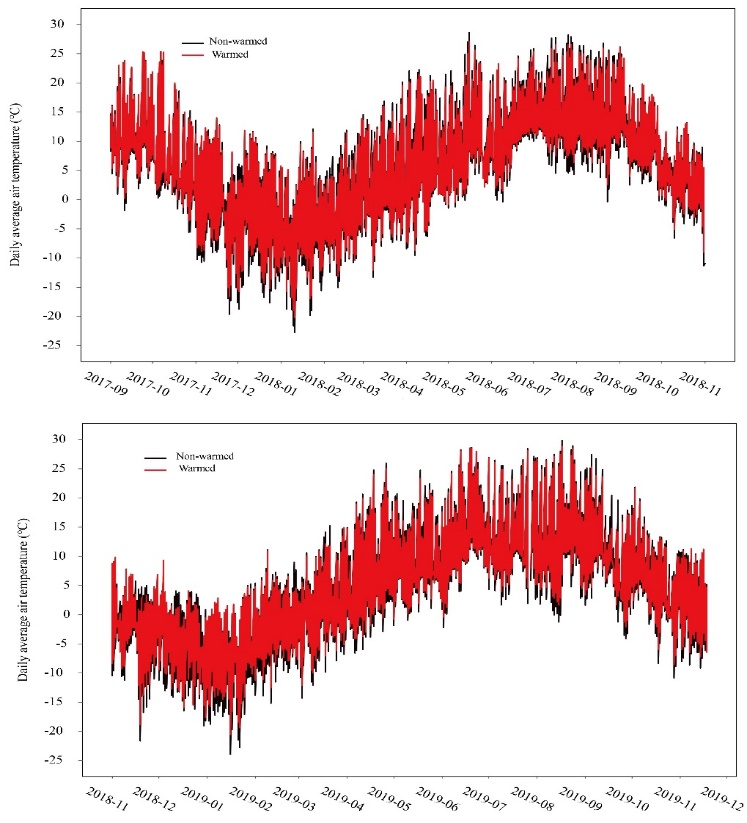


**Figure S3** Monthly average soil temperature at -5cm in the non-warmed and warmed treatments from 2015 to 2019.


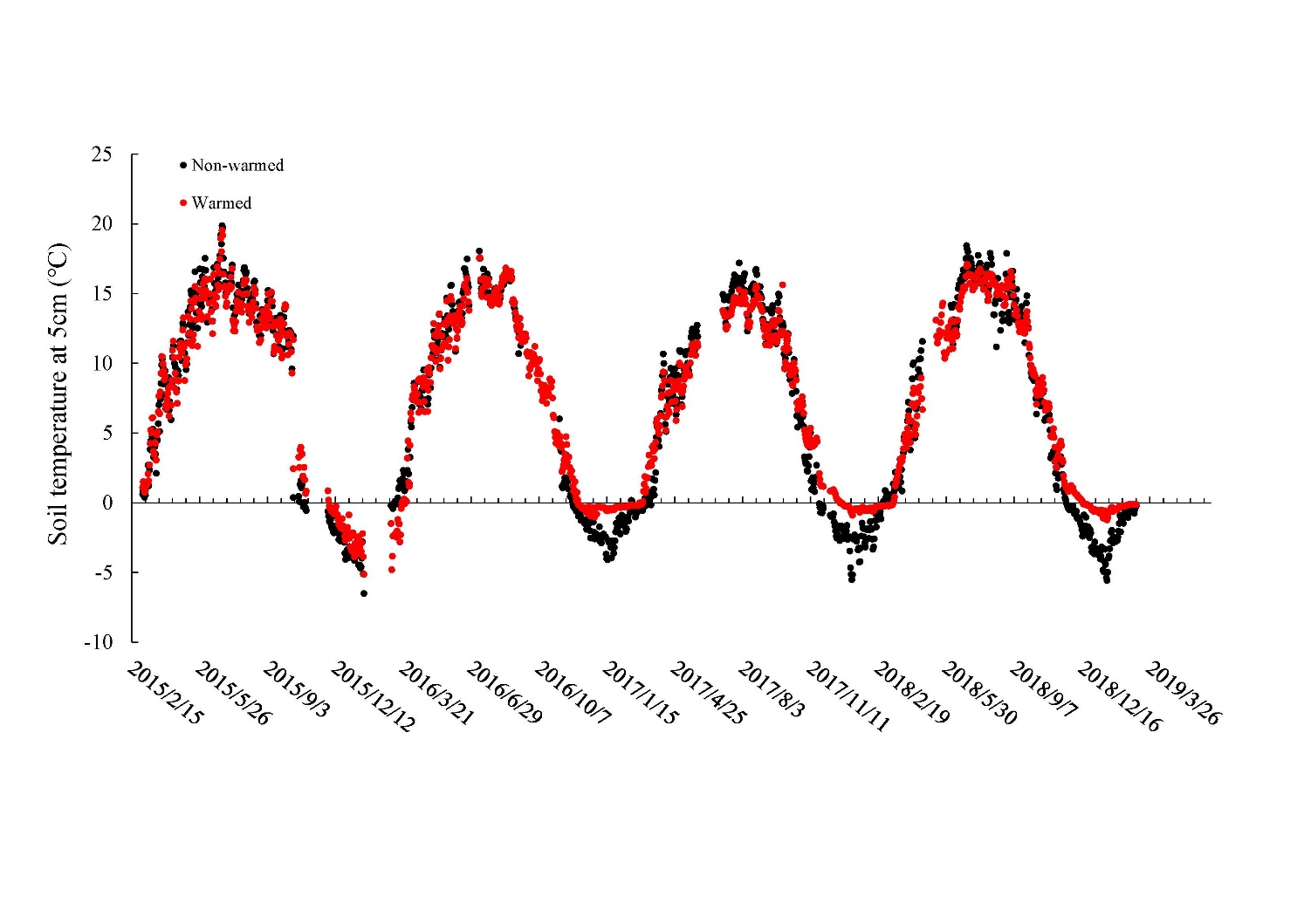


**Figure S4** Variation in temperature at 30cm above ground in the non-warmed and warmed treatments during 24 hours of April, July, October, and January in 2018 and 2019. *, *P* < 0.05; **, *P* < 0.01; ***, *P*< 0.001.


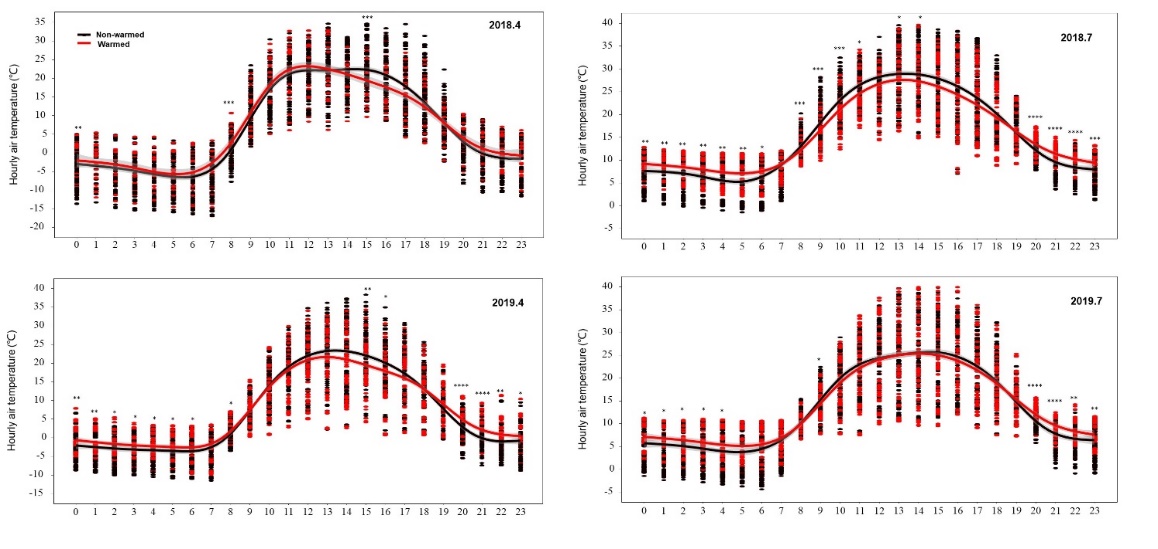


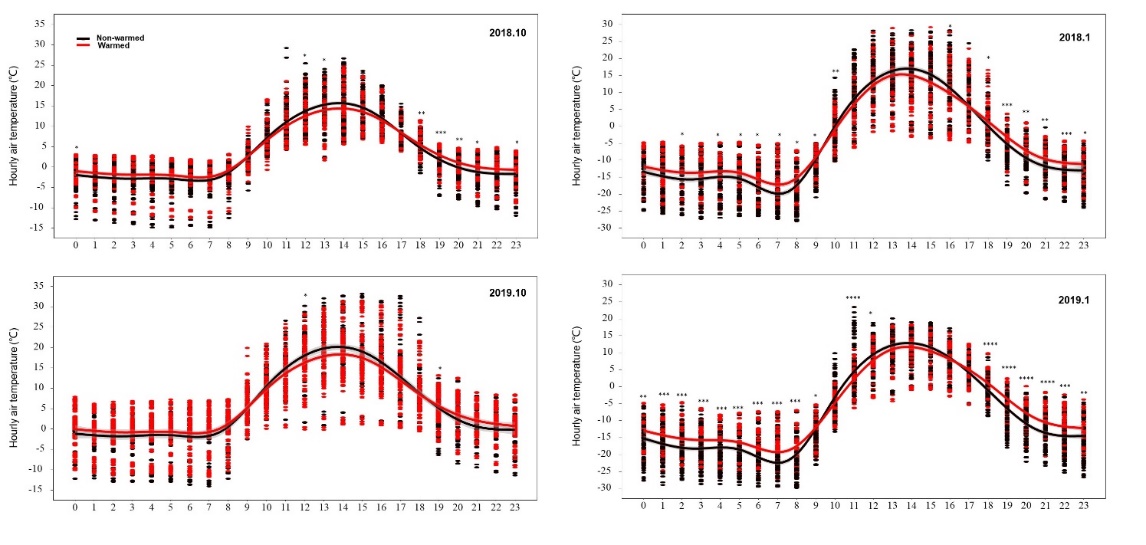


**Figure S5** Difference in temperature at 30cm above ground in the non-warmed and warmed treatments between day and night from September 2017 to November 2019. *, *P* < 0.05; **, *P* < 0.01; ***, *P*< 0.001.


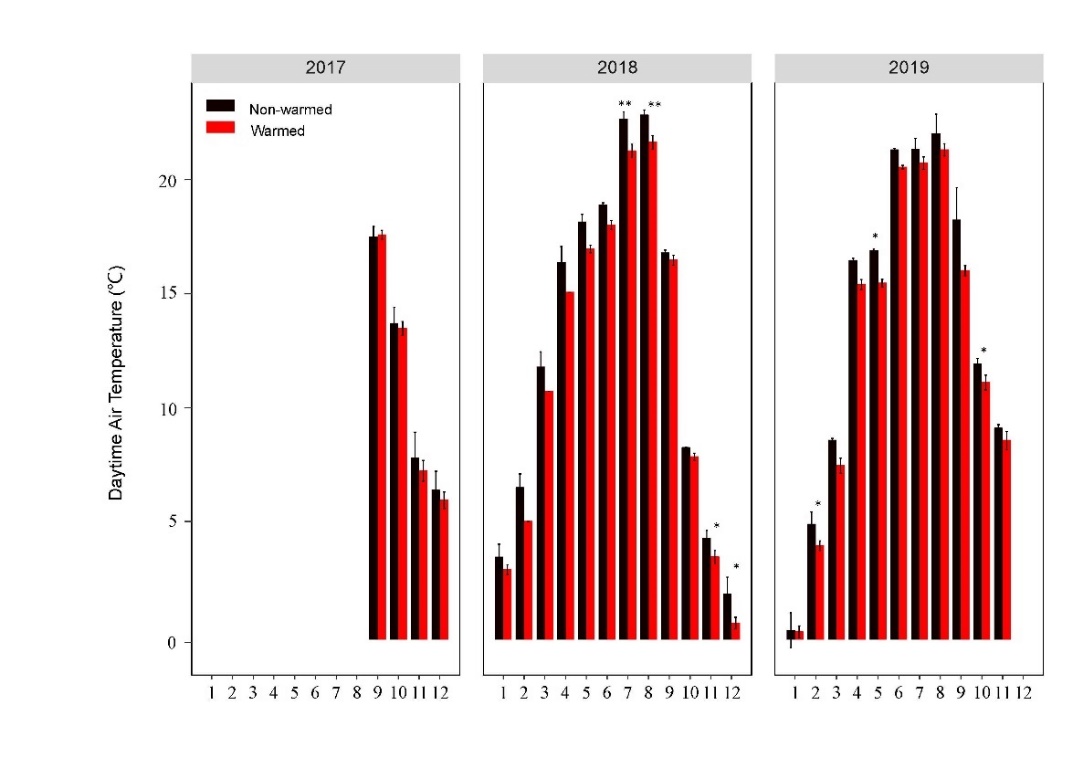

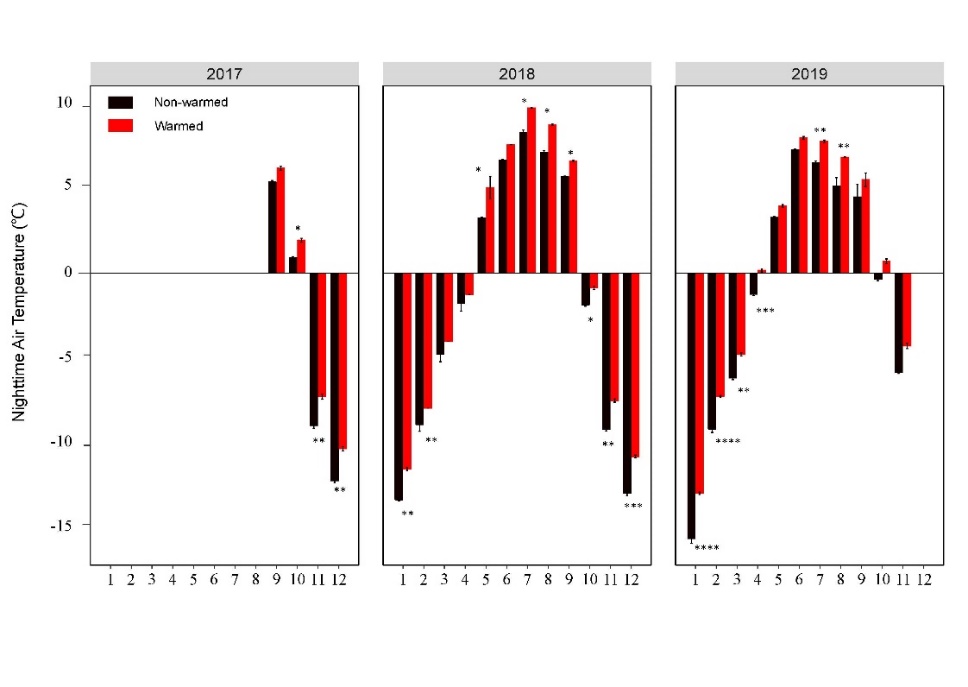


**Figure S6** Monthly average air vapor pressure deficit (VPD) above 30cm ground surface in the non-warmed and warmed treatments from May 2018 to October 2019.

**
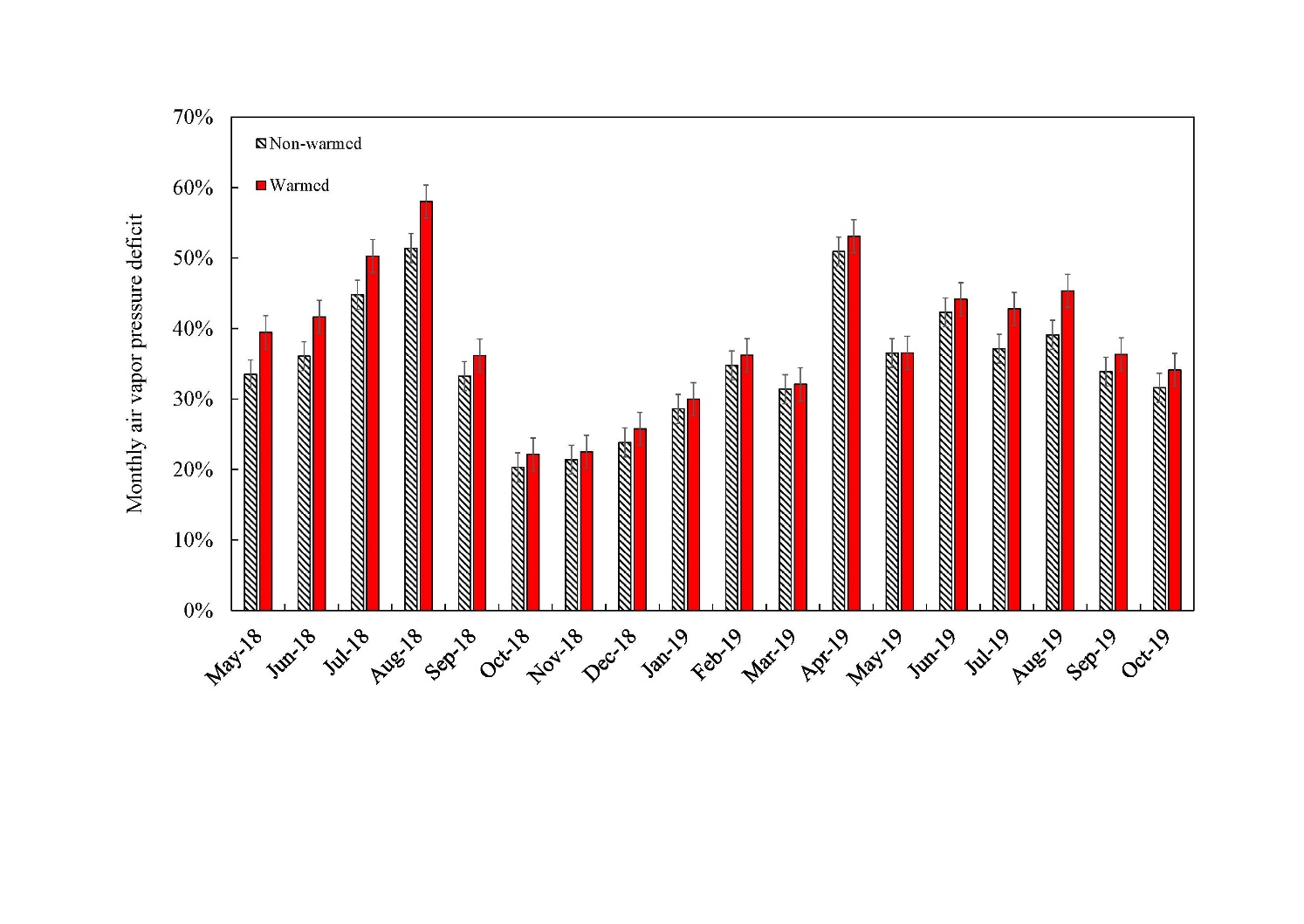
**

**Figure S7** Monthly average volumetric soil water content (VWC) at -5cm during the growing season from May to September in the non-warmed and warmed treatments from 2015 to 2019.


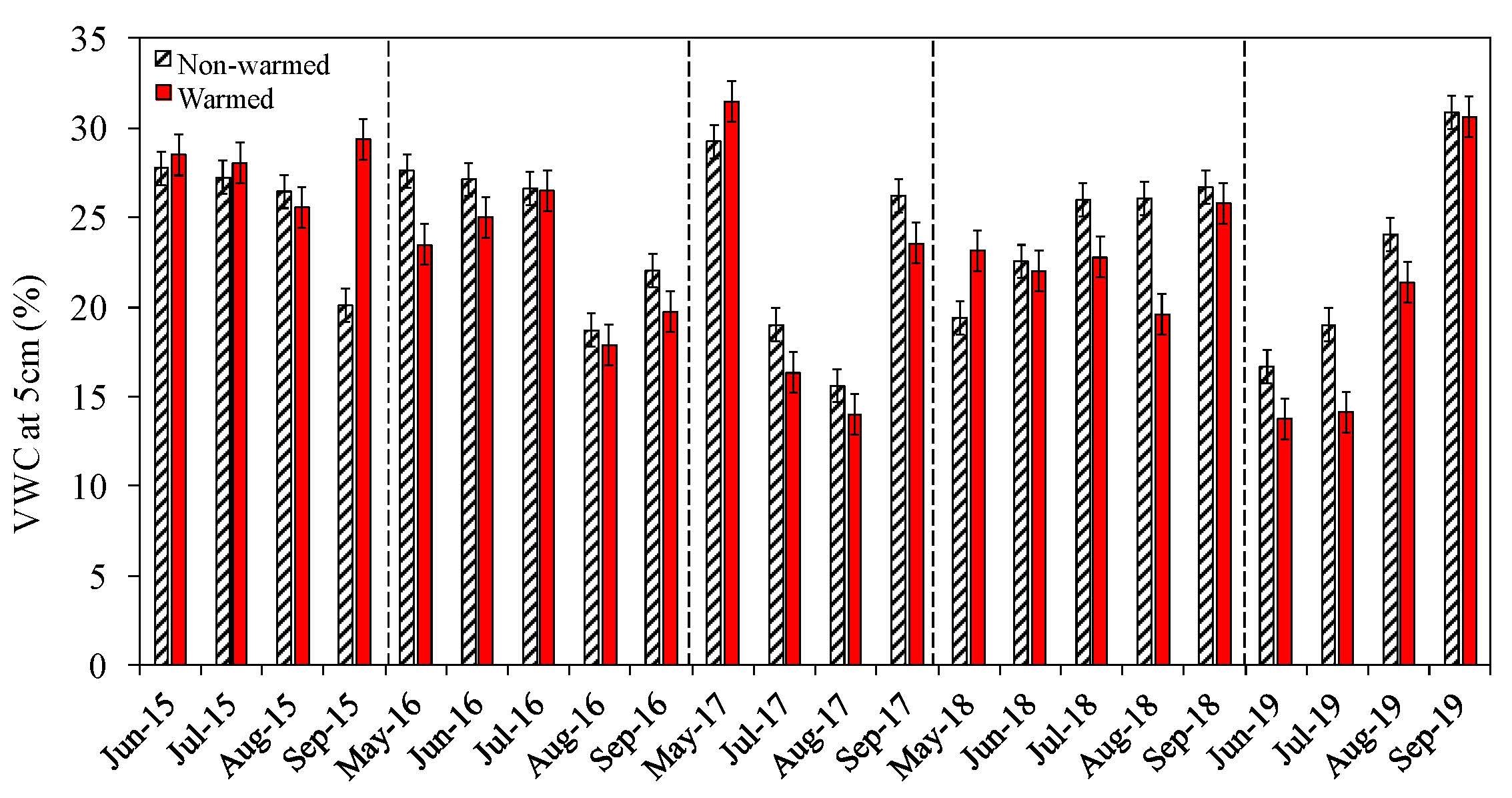

Supplement: Supplementary file 1 [file Table_1.docx]
